# Dominant bacterial taxa drive microbiome differences of juvenile Pacific oysters of the same age and variable sizes

**DOI:** 10.3389/frmbi.2023.1071186

**Published:** 2023-03-30

**Authors:** Mary K. English, Chris J. Langdon, Carla B. Schubiger, Ryan S. Mueller

**Affiliations:** ^1^ Department of Microbiology, College of Science, Oregon State University, Corvallis, OR, United States; ^2^ Department of Fisheries, Wildlife, and Conservation Sciences, College of Agricultural Sciences, Oregon State University, Corvallis, OR, United States; ^3^ Department of Biomedical Sciences, Carlson College of Veterinary Medicine, Oregon State University, Corvallis, OR, United States

**Keywords:** oyster microbiome, aquaculture, *Mycoplasma*, *Crassostrea gigas*, spat development

## Abstract

Oyster aquaculture is a growing industry that depends on production of fast-growing, healthy larvae and juveniles (spat) to be sold to farmers. Despite nearly identical genetics and environmental conditions in the early life stages of oysters, larvae and spat sizes can vary drastically. As the microbiome can influence the health and size of marine invertebrates, we analyzed the microbiomes of differently-sized juvenile Pacific oyster (*Crassostrea gigas*) spat of the same age to examine the relationship of their microbiomes with size variation. We used 16S sequencing of 128 animals (n = 60 large, n = 68 small) to characterize the microbiomes of each size class, comparing alpha diversity, beta diversity, and differentially abundant taxa between size classes. We observed that small spat had higher alpha diversity using measures that considered only richness, but there was no difference in alpha diversity between the two size classes using measures that incorporate compositional metrics. Additionally, large and small spat had distinct microbiomes, the separation of which was driven by more dominant bacterial taxa. Taxa that were differentially abundant in large oysters were also more abundant overall, and many appear to have roles in nutrient absorption and energy acquisition. The results of this study provide insight into how the microbiome of *C. gigas* may affect the early development of the animal, which can inform hatchery and nursery practices.

## 1 Introduction

In 2018, aquaculture facilities in the United States produced over 1.5 billion USD in seafood, with more than half of this amount coming from shellfish production (NOAA, 2021). Oysters have been the highest-producing marine shellfish by value in recent years in the USA, accounting for almost 220 million USD in 2018. Bivalve hatcheries and nurseries produce larvae and juvenile oysters (spat), respectively, that are planted on farms for cultivation and harvest (Prado et al., 2010). Substantial size variation is commonly observed among juvenile shellfish (Lemos et al., 1994; Collet et al., 1999). Small, slower-growing animals are culled by size-selective sieving (Bardach et al., 1974), leaving only a fraction of the animals available for farms (Taris et al., 2006). It is in the interest of aquaculture that the causes of this size variation are better understood to reduce losses and maximize production efficiencies.

Research has shown that genetic factors are correlated with size variation (Singh and Zouros, 1978; Ernande et al., 2003), but while it is known that the microbiome is correlated with the health and growth in marine finfish (Forberg et al., 2016; Trinh et al., 2017), little work has been done exploring the relationship between variation in oyster growth rate and microbiome composition and function. Given the relationship of the microbiome and host performance, we hypothesize that the microbiome may help explain some of the observed variations in growth rates and sizes of spat that are of similar genetic origin and raised under the same conditions.

Oysters and other filter-feeding bivalves accumulate microbes from their environment while feeding, ingesting free-living and food particle-associated bacteria (Langdon and Newell, 1989; Kreeger and Newell, 1996; Cranford et al., 2011). Bacteria in the gut of oysters have been reported to be present at densities tenfold greater than in the surrounding seawater (Cavallo et al., 2009). As a result, a resident microbiome forms with the highest bacterial density in the digestive system and lower densities in other parts of the oyster (Kueh and Chan, 1985). Bacteria colonize the gastrointestinal tract, becoming permanent residents over time if they are able to maintain their presence in the gut and proliferate (Verschuere et al., 2000). ​​During early growth stages, the microbiome of *C. gigas* plays an important role in immune system development (Fallet et al., 2022). Studies also suggest that the microbiomes of marine invertebrates can be assembled in ways that match the predominant diet of the host, enhancing nutrient extraction from specific food sources. For instance, abalone (*Haliotis midae*) host saccharase-producing bacteria in the gut, matching the animal’s saccharide-based seaweed diet (Erasmus et al., 1997).

The oyster microbiome community structure varies by extrinsic factors, such as geography and temperature, and intrinsic factors such as host age, genetics, and health. The microbiome is known to be associated with host health and disease in aquaculture. For example, King et al. (2019a) found that Pacific oysters had distinct microbiomes enriched in certain taxa depending on an oyster’s susceptibility to ostreid herpesvirus (OsHV-1). Relationships between animal microbiomes and growth have been found in many aquaculture species, including juvenile killifish (Forberg et al., 2016), Atlantic cod (Forberg et al., 2016; Trinh et al., 2017), Pacific white shrimp (Fan et al., 2019), black tiger shrimp (Infante-Villamil et al., 2019; Duan et al., 2020), and razor clams (Dai et al., 2022). The oyster microbiome has been explored with regard to animal age, environmental influences, and the relation to health and disease (Trabal Fernández et al., 2014; Lokmer and Wegner, 2015; Pierce et al., 2016; Green et al., 2019; King et al., 2019b; Scanes et al., 2021). We performed an observational study to determine if differences in microbiome were correlated with differences in juvenile oyster (spat) size while holding their genetics, age, culture environment, and diet constant. We also identified microbial taxa that may affect spat growth in aquaculture settings.

## 2 Materials and methods

### 2.1 Sample collection

*C. gigas* larvae were produced in March 2020 by crossing gametes of selected parents of known pedigree to produce families of cohort 29 of the Molluscan Broodstock Program at Oregon State University. Oysters were grouped by “family”, defined as a cohort of animals from the same parents and reared under the same conditions. The larvae of each family were raised to metamorphosis in separate cultures, resulting in the production of spat, as described by Langdon et al. (2003). Spat from each family were separately reared in upweller containers in a land-based nursery that was continuously supplied with sand-filtered seawater at 18 °C. The spat were fed *ad libitum* on a mixed algal diet of flagellate *Isochrysis galbana* (C-ISO strain) and the diatoms *Chaetoceros muelleri* and *C. gracile*. The culture system was cleaned with a jet of pressurized freshwater every day. When the spat reached 3 to 5 mm, families were transferred to separate labeled PVC mesh bags at 400 spat per bag and placed in outdoor tanks where they were fed the same mixed algal diet and constantly supplied with sand-filtered seawater at ambient temperatures of 10-12 °C. Growth was limited by batch-feeding the oysters only once or twice a week with the seawater flow turned off until the algal ration had been consumed during each feeding period. The culture system and spat bags were cleaned every week using pressurized freshwater, and spat bags were shaken to re-distribute spat within the bags. In October 2020, spat were sampled after seven months of culture. They were not fed in the 48 hours before collection to reduce the effects of recently consumed particulate material on gut flora composition. Ten of the visually largest and ten of the smallest oysters in each family were removed from mesh bags. Animals were briefly submerged in 100% ethanol, patted dry, and put into 50 mL vials containing 100% ethanol. They were stored at -20 °C; within 4 hours of the start of sample collection and processed within six months.

### 2.2 DNA extraction, PCR, and sequencing

Eight large and eight small oysters from each of ten families were selected for microbiome sequencing, except for family 94, in which all ten large and small oysters were extracted for microbiome sequencing (though only eight were ultimately sequenced). The remaining two sampled oysters from each size class and family were used to determine spat weights (see “Processing and weighing samples”). For microbiome sequencing, each animal was shucked by prying open the shell hinge with a sterile scalpel in a sterile Petri dish. The soft tissue was separated from the shell, cut into 1 mm pieces, and placed in a 2 ml tube containing lysing matrix A and a 35 mm ceramic bead (both MP Biomedicals, Santa Ana, CA, USA) and 1 ml CTAB buffer (Crump et al., 2003). A phenol:chloroform method was used to extract DNA (as in Crump et al., 2003, except that RNAse was added after sodium dodecyl sulfate). DNA pellets were dried in a roto-vac and resuspended in 50 µl molecular grade water (Thermo Fisher Scientific, Waltham, MA, USA). DreamTaq green master mix (Thermo Fisher Scientific, Waltham, MA, USA) was used to amplify 16S V4 hypervariable locus following the manufacturer’s instructions and with the following cycles: A 3 minute initial denaturation at 94 °C; 30 cycles of 45 second denaturation at 94 °C, 1 minute anneal at 52 °C, and 1.5 minute extension at 72 °C; a 10 minute final extension at 72 °C; and a hold at 4 °C. Modified dual-indexed 515F and 806R universal 16S rRNA (V4) primers were used based on Kozich et al. (2013) and modifications were based on Wang et al. (2021). Amplicon quantities and sizes were checked with agarose gel electrophoresis. All but seven (147/154) samples were amplified successfully. Agencourt AMPure XP beads (Beckman Coulter, Brea, CA, USA) were used to purify the PCR reactions per the manufacturer’s instructions. A Qubit 2.0 fluorometer (Thermo Fisher Scientific, Waltham, MA, USA) was used to quantify concentrations of purified amplicons, and these values were used to pool libraries to an equimolar concentration prior to paired-end 2 x 250 bp sequencing with the Illumina MiSeq V2 system (Illumina Inc., San Diego, CA, USA). Samples were demultiplexed by the Center for Quantitative Life Science (CQLS) at Oregon State University.

### 2.3 DADA2 sequence analysis

The DADA2 package (v. 1.20.0; Callahan et al., 2016) was used to analyze raw sequencing reads of 16S amplicons within the R environment (v. 2021.9.0.351; RStudio Team, 2021). The ‘filterandtrim’ command with default settings and truncation lengths based on FASTQC score reports was used to quality filter the raw sequences. Default settings were used for error-rate training, denoising, and paired-read merging to create representative amplicon sequence variants (ASVs) and create a sequence count table of ASVs. Due to uneven sequencing depth that ranged from 100s to ~65,000 reads per sample, we set upper and lower read depth limits. To determine an appropriate sequencing depth range to capture all observed taxa, the ‘rarecurve’ function of the vegan R package (v. 2.5.7; Okansen et al., 2019) was used to make a rarefaction curve. Based on this curve, only samples with a minimum depth of 5000 reads were kept, with 128/147 libraries remaining (**Table S1** and **Figure S1**). A maximum of 15000 reads per sample was chosen as an upper limit, and libraries over 15000 reads were rarefied using the ‘rrarefy’ function of vegan. The number of reads per sample, which ranged from 5000 to 15000, is referred to as the rarefied sampling depth. ASVs were assigned to taxonomic rankings down to the genus level using a naive Bayesian classifier (Wang et al., 2007) trained on the SILVA database (v. 132; Quast et al., 2013). A total of 3181 unique ASVs across 128 samples remained after removing ASVs assigned as mitochondria or chloroplasts. ASVs contributing < 0.1% of the total reads across all libraries were excluded from subsequent steps except for the core microbiome analyses, leaving 97 ASVs for community analysis.

### 2.4 Microbiome community analysis: Alpha and beta diversity

To make a phylogenetic tree containing the ASVs, the ‘align.seqs’ and ‘filter.seqs’ commands of the mothur software package (v.1.39.3; Schloss et al., 2009) were used to make filtered alignment of the ASV sequences against the pre-computed SILVA Ref NR 132 alignment. FastTreeMP (v. 2.1.11 SSE3; Price et al., 2009) was used to calculate a phylogenetic tree from the alignment using a generalized time-reversible model of evolution, and rerooted at its midpoint using ‘reroot.pl’ (Junier and Zdobnov, 2010). The ASV table, taxonomy table, sample metadata (including size category, spat family, and dry weight), filtered sequences, and phylogenetic tree were combined to make a phyloseq object (v. 1.23.0; McMurdie and Holmes, 2013). Richness, Simpson, and Shannon alpha diversity indices were calculated using the ‘estimate_richness’ function in phyloseq. Richness is the number of observed species, with no regard to their evenness (relative abundance). The Simpson index (Simpson, 1949) weighs species evenness higher than richness (Wagner et al., 2018). The Shannon index (Shannon, 1948) equally weighs richness and evenness (Wagner et al., 2018). Faith’s phylogenetic diversity, which is a richness measure calculated by examining relatedness of taxa without considering relative abundance (Faith, 1992), was calculated using the ‘PD’ function in picante (v. 1.8.2; Kembel et al., 2010). The four alpha diversity scores were normalized to compare between indices using phyloseqCompanion (Stagaman, 2022). If assumptions of normality were not met, then generalized linear models (GLMs) were used in the ‘stats’ package (v. 4.1.1; R Core Team, 2022). Beta diversity measured with weighted UniFrac metrics (Lozupone et al., 2011) to account for taxa relatedness within a sample was calculated using the count table and phylogenetic tree in vegan. A PERMANOVA test for significant effects of spat family and size on clustering was performed using the ‘adonis2’ function in vegan. Family was treated as a random effect using the setBlocks option for permutations. To test for differences in variance, the ‘betadisper’ function in vegan was used. All plots were created with the ‘ggplot2’ package in R (v. 3.3.5; Wickham, 2016). Reported summary statistics are mean ± standard deviation unless otherwise stated.

### 2.5 Core microbiome

All 3181 amplicon sequence variants, their accompanying filtered sequences, and phylogenetic tree were used to create a phyloseq object to define the core microbiome. To investigate whether oysters of different size classes harbored distinct microbiomes, the main phyloseq object was subset into two objects based on size class (large and small; n = 60 and 68 samples, respectively). Due to small sample sizes within families by size class (n = 4 to n = 8 replicates per size class per family), core microbiomes were not investigated for each family and size. Subsets were made of each phyloseq object similar to a previously established method (Ainsworth et al., 2015), wherein the number of ASVs found in an increasing number of samples was plotted (1 to 100% of samples, in increments of 1%). The core microbiome threshold was defined as the number of ASVs that remained constant over a 4% increase in the number of samples in a size class.

### 2.6 Differential abundance

The ANCOMBC package (v. 2.0.2; Lin and Peddada, 2020) was used to detect ASVs that were differentially abundant in one size class over the other. The ‘ancombc2’ function was run in R with default settings, with two exceptions: the Benjimini Hochberg method was used to adjust p-values, and ‘neg_lb’ was set to TRUE following developer’s recommendations when using large sample sizes. The ‘fix_formula’ option was set to incorporate the covariate of spat family (Size + Family), as recommended by the developers. Only ASVs with an adjusted p-value of ≤ 0.05 were classified as differentially abundant. We have reported log fold change (LFC), the magnitude of differential abundance in large spat over small from ANCOM-BC2’s natural log model. To verify that there was no interactive effect between differentially abundant ASVs and each family, a second model testing for differential abundance between large and small spat was created with ‘fix_formula’ set to an interactive effect (Size * Family). All other parameters were the same as the first run.

### 2.7 Processing and weighing samples

Each family and size class had at least two leftover animals that were not used for microbiome extraction, except for family 94, which had DNA extracted from all ten of each size class (though only eight were sequenced). These 36 oysters were patted dry and weighed for wet weight when the shells were closed. For dry weight, oysters were split open with a scalpel and dried at 60 °C; for 48 h. For both wet and dry weights, the measurement included both tissue and shell. For all subsequent weight-based analyses, the averages of the two dry weights for each family and size class were assigned as representative weights for all animal samples of that family and size class. The ‘descdist’ function in the ‘fitdistrplus’ package (v. 1.1-8; Delignette-Muller and Dutang, 2015) in R was used to fit the distribution of dry weight as quasibinomial. The ‘glm’ function in the ‘stats’ package was used to fit a generalized linear model to test for weight differences based on family assignment. All reported weights are mean ± standard deviation.

## 3 Results

### 3.1 Oyster size

**Figure 1:**
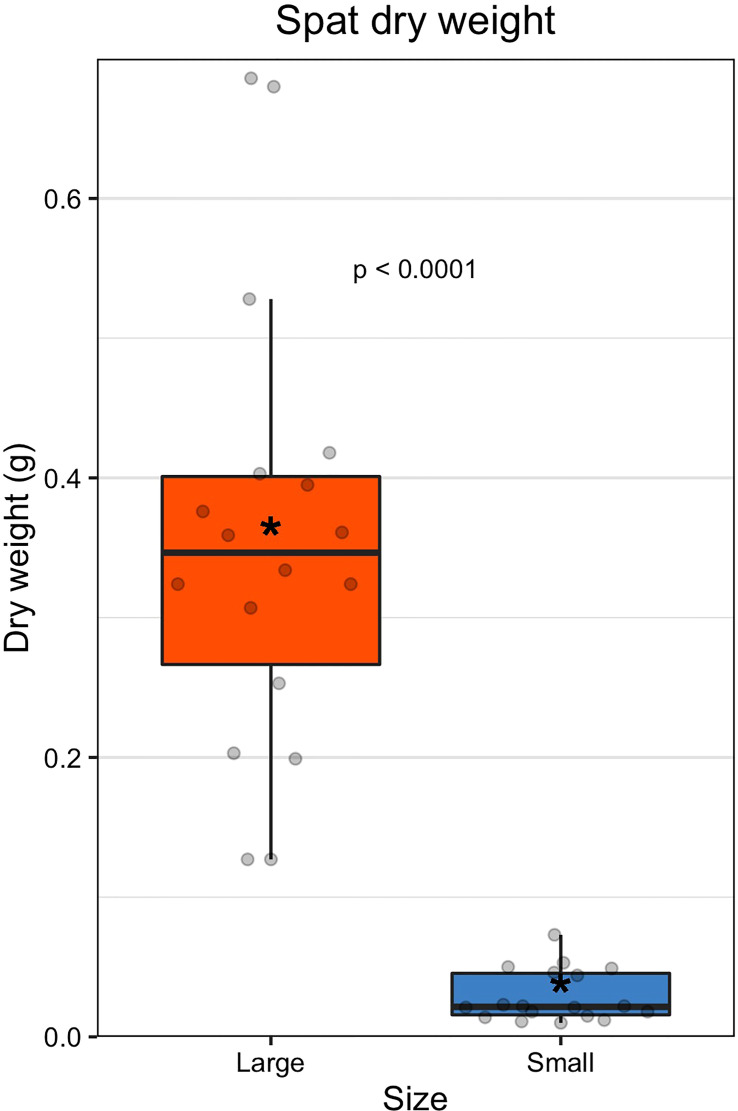
Dry weight of spat by size class. The colored boxes represent upper and lower quartiles, horizontal lines in boxes show the median values, and the vertical whiskers represent the most extreme values within the 1.5x interquartile range. Asterisks display mean values.

Large oysters were on average 12-fold larger than small oysters by dry weight (large = 0.356 ± 0.137 g; small = 0.029 ± 0.018 g; **Figures 1**, **S2**, **S3**, and **Table S2**). The size classes were statistically different by dry weight (GLM: β = 2.917, 34, p < 0.0001). Family did not have a significant effect on dry weight (ANOVA: F(8,27) = 0.47, p = 0.867).

### 3.2 Overall community structure of oyster microbiomes

A total of 97 taxa represented 99.9% of all sequence reads. Communities were generally skewed in abundances of dominant taxa, with few taxa representing most of the reads and many low-abundance taxa constituting the rest. The two most abundant ASVs in the dataset, with ASV01 accounting for 25.07% and ASV02 accounting for 6.51% of all reads, belong to the genus *Mycoplasma* in the class Mollicutes. The remaining 95 taxa were present at relatively low abundances (< 5% of total reads; **Table S3**).

### 3.3 Alpha diversity

**Figure 2:**
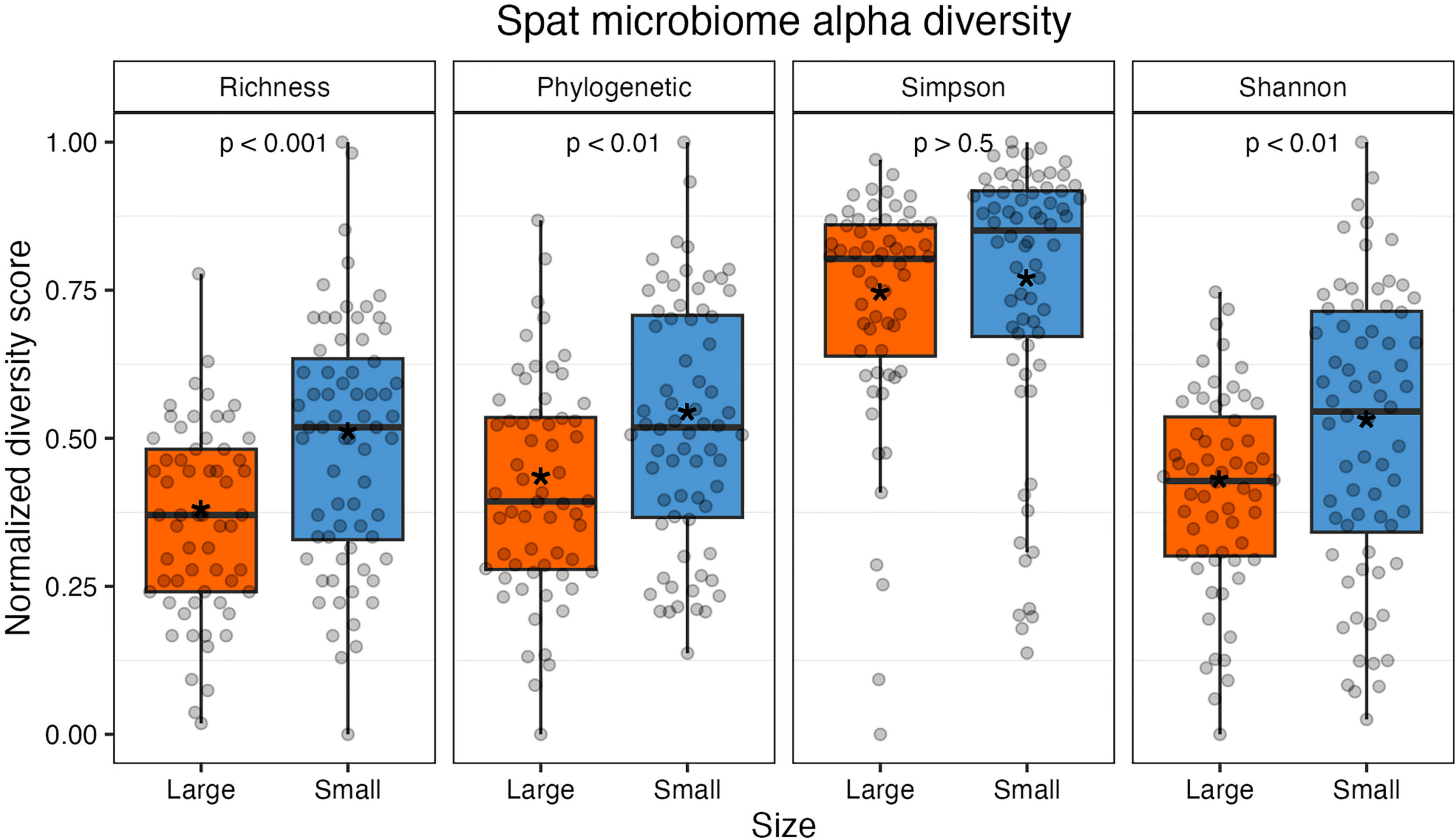
Alpha diversity measures normalized from 0 to 1 (from left: richness, Faith’s phylogenetic distance, Simpson index, and Shannon index). Individual samples are represented by gray points. All indices except Simpson were statistically significant between size classes. The colored boxes represent upper and lower quartiles, horizontal lines in boxes show the median values, and the vertical whiskers represent the most extreme values within the 1.5x interquartile range. Asterisks display mean values.

After filtering out rare taxa (see methods), alpha diversity metrics were calculated using the remaining 97 ASVs; due to this additional filtering step, alpha diversity metrics here are a conservative estimate of sample richness. Small oysters had higher average values in richness, phylogenetic distance, and Shannon index than large ones (**Figure 2** and **Table S4**). Richness and phylogenetic indices, which are two measures that consider evenness, were both statistically higher in small spat (richness, t(123.64) = -4.0, p < 0.001; phylogenetic, t(126) = -3.1, p < 0.01). The Shannon index, which depends on both richness and evenness of taxa in a sample, was significantly different by size (t(119.5) = -2.77, p < 0.01); however, the Simpson index, which weighs dominance more heavily than the Shannon index, was not significantly different by size (GLM: β = 0.13, 126, p = 0.53). It can then be inferred that it is the richness, or changes in the presence and absence of rare, low abundance taxa, that drives the observed difference in Shannon indices between large and small oysters. When removing low abundance taxa (for instance, including only taxa that account for 98% of all reads), the Shannon index is no longer significantly different between sizes (t(124.94) = 0.31, p > 0.5; **Figure S4**).

Oyster family did not have a significant effect on Shannon or Simpson index (p > 0.05) but did have a significant effect on phylogenetic distance and richness (p < 0.05). Rarefied sampling depth did not impact diversity for Shannon or Simpson indices (p > 0.05) but did impact phylogenetic distance and richness metrics (p < 0.05; **Table S5** and **Figure S5**).

### 3.4 Beta diversity

**Figure 3:**
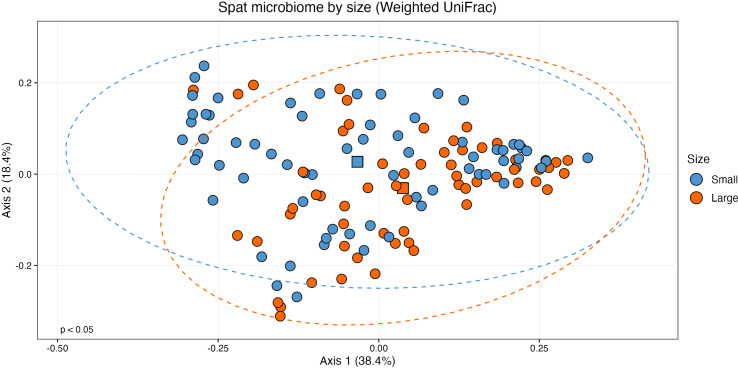
Principal coordinates analysis (PCoA) plot of beta diversity based on weighted UniFrac distances. Samples are grouped by size class (n = 60, large; n = 68, small) with statistically distinct centroids (PERMANOVA: F(1, 126) = 2.532, p < 0.05). Ellipses show 95% confidence intervals of the centroid estimates of the community structures of each size class. The centroid of each size class is shown as a square.

When considering size class, the centroids for small and large samples were statistically distinct on a PCoA plot of weighted UniFrac distances (**Figure 3**; PERMANOVA: F(1, 126) = 2.532, R^2^ = 0.0197, p < 0.05). Variance was also statistically different between the two sizes (ANOVA: (F(1,126) = 5.2, p < 0.05). As variance and centroid significance cannot be partitioned, the significant p-value from the centroid PERMANOVA test reflects both different variance and centroids. There were no significant effects of oyster family (PERMANOVA: F(9, 118) = 1.432, R^2^ = 0.0985, p = 0.056) or rarefied sampling depth (PERMANOVA: F(1,126) = 0.930, R^2^ = 0.0073, p = 0.435) on community structure.

### 3.5 Core microbiome by size class

**Figure 4:**
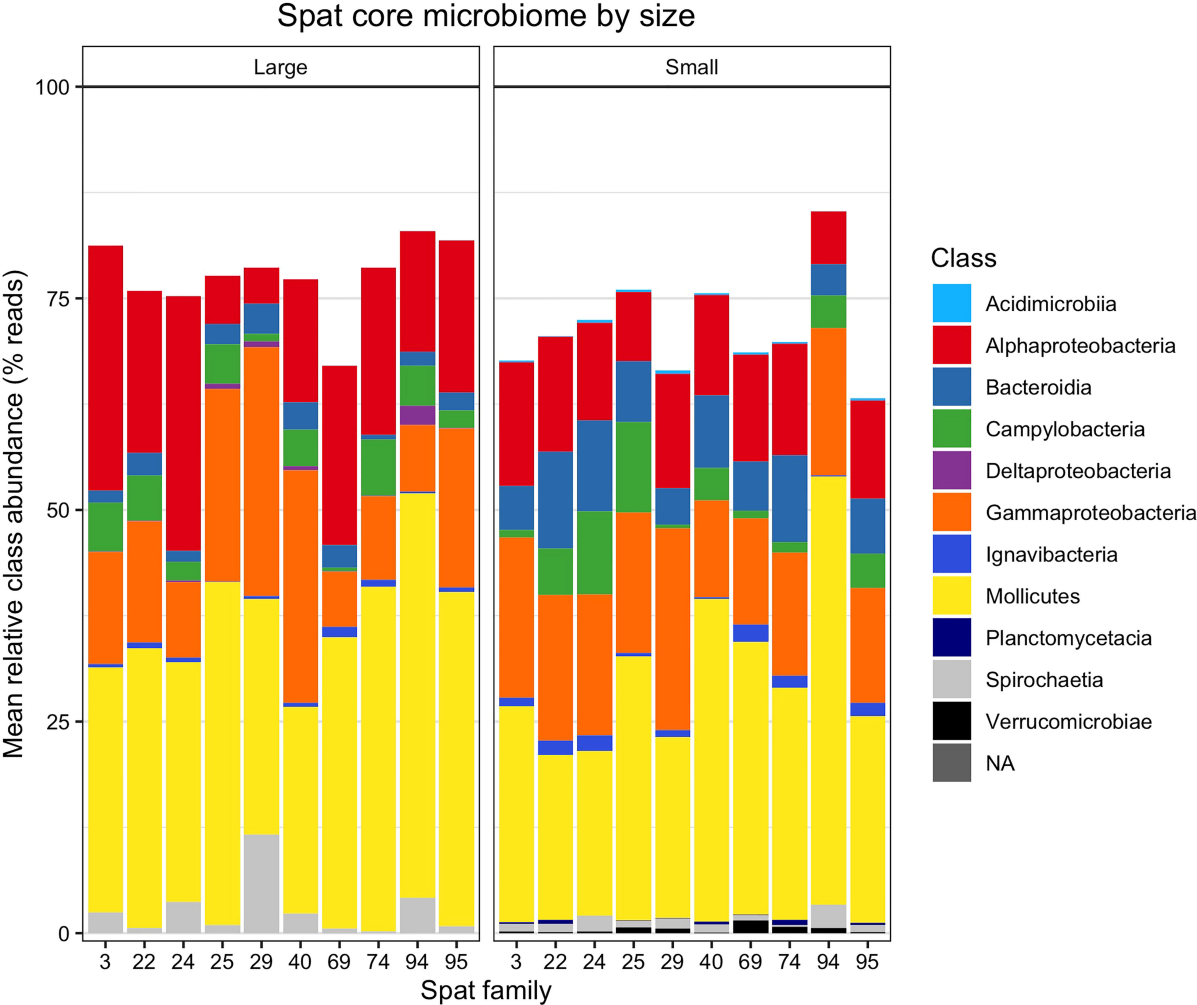
Relative abundances of taxonomic classes within the core microbiomes of large and small spat. Relative abundance is averaged by size and oyster family. Each stacked vertical bar represents one oyster family and size class, consisting of 4-8 replicate oysters sampled per family and size class. Abundance does not sum to 100% because non-core microbiome members are omitted.

The number of ASVs present in samples began to level out at 52% of samples for large oysters, and 56% for small oysters (**Figure S6**), resulting in a subset of 26 ASVs in the large core microbiome (**Table S6**) and 35 ASVs in the small core microbiome (**Table S7**). ASV01, *Mycoplasma* sp. in the class Mollicutes, was the only ASV present in every sample; by comparison, the second-most abundant ASV (ASV02, also *Mycoplasma* sp.) was only present in 85.9% of samples. The small and large core microbiomes shared 19 of these ASVs, meaning seven were present only in the large core microbiome, and 16 were present only in the small core microbiome (**Figure 4**).

### 3.6 Differential abundance

**Figure 5:**
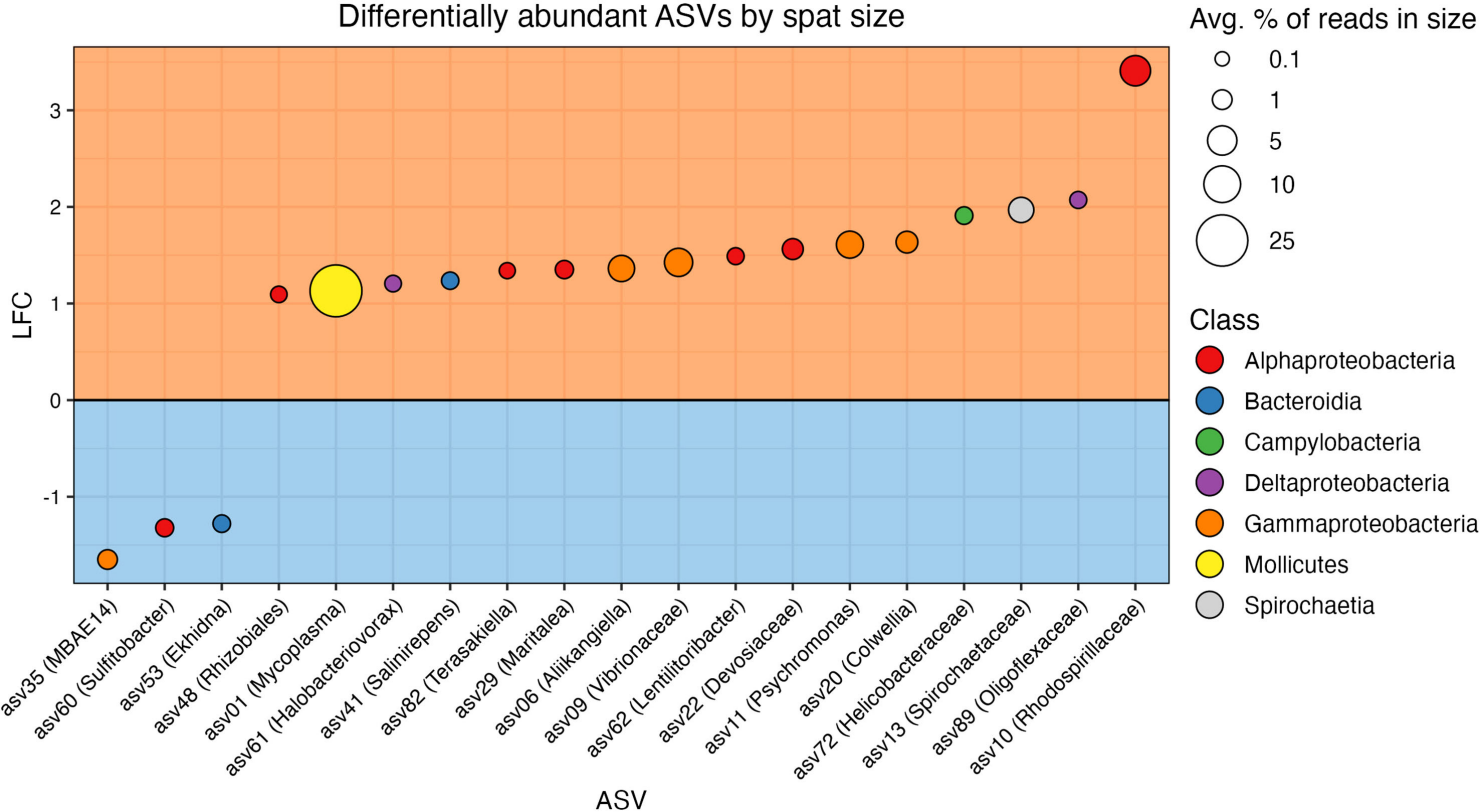
Results from ANCOM-BC2 showing differentially abundant ASVs in each size class. ASVs are colored by taxonomic class. The orange box (top) includes ASVs differentially abundant in the large spat, and the blue box (bottom) includes ASVs differentially abundant in the small spat. Vertical axis shows log fold change (LFC), the magnitude of differential abundance in large spat over small (Lin and Peddada, 2020). The size of points corresponds to the relative abundance of the ASV in the size class in which it is differentially abundant.

A total of 19/97 taxa were differentially abundant between size classes (**Figure 5** and **Table S8**). Three ASVs were found in higher relative abundance in the small oysters, while 16 were detected in higher relative abundance in the large. The most common taxonomic group over-represented in the large oysters was Proteobacteria, with 12 of the 16 ASVs belonging to this phylum (6 Alphaproteobacteria, 4 Gammaproteobacteria, which includes one Vibrionaceae, and 2 Deltaproteobacteria). Alternatively, one ASV each of the Alphaproteobacteria, Gammaproteobacteria, and Bacteroidia classes were the three taxa that were more abundant in small oysters. Most of the differentially abundant ASVs in each size class (12/16 in large, and 3/3 in small) were also members of their respective core microbiomes. When testing for interactive effects between spat family and ASV differential abundance, there were no significant q-values that would indicate an interaction.

## 4 Discussion

Oysters harbor bacterial communities that are known to be correlated with the organism’s health and vary based on host genetics, age, rearing environment, and feed. Studies have established a strong relationship between oyster size and development in terms of sexual maturity (Pouvreau et al., 2000; Akélé et al., 2017). Our study shows that there is a relationship between the microbiome and the size of *C. gigas* spat. Small oysters showed higher alpha diversity by most measures, suggesting that the oyster undergoes a microbial community succession with size, which we infer to be related to development, and that the loss of certain low-abundance taxa may be a part of a selective process initiated by the host.

Other aquaculture studies have noted a reduction in alpha diversity that correlates with organism age. Trabal Fernández et al. (2014) found higher gut microbiome diversity in postlarval life stages of three different oyster species, including *C. gigas*, compared to adults. The postlarval individuals had higher richness based on observed operational taxonomic units (OTUs) and the Chao1 estimate, which emphasizes less abundant taxa, and higher Shannon and Simpson indices, both of which consider the relative abundance of taxa in a sample. The reduction in alpha diversity with size suggests that the conditions in the gut become more selective for a less rich microbiome as the animal grows. This pattern has also been observed in other marine species, such as cod larvae and killifish. Forberg et al. (2016) found that richness, but not Shannon diversity, was higher for small compared to large killifish of the same age. The same study found that small and large cod larvae did not vary in richness, but small larvae had a higher Shannon index than large larvae. Arfken et al. (2021) compared larval *C. virginica* oysters at different early stages of development and observed a decrease in richness with age; however, they did not detect a difference in the Shannon index. Contrastingly, Duan et al. (2020) compared postlarval shrimp of the same age, genetic background, and rearing conditions that differed in growth rate in an experimental approach that was very similar to ours. No significant difference in richness was observed between size classes. Dai et al. (2022) found statistically higher alpha diversity in the clam gut microbiome measured by the Shannon index in the largest compared to the smallest clams, but no difference in the richness was observed. The inconsistencies between our study and the two similar studies carried out with clams and shrimp may be attributed to inherent differences among host organisms. Combined with observations that the gut microbiome of bivalves is consistently different from its environment (Valley et al., 2009; Lokmer and Wegner, 2015), the phenomenon of alpha diversity reduction with age and size suggests that there is a degree of selection of microbial community occurring during animal development. This selection with age has also been proposed in Atlantic salmon (Bozzi et al., 2021).

Each size class of spat harbored a statistically significantly different microbiome, while each spat family did not. These results agree with the study of Dai et al. (2022) examining razor clam microbiome variation among different-sized individuals. Dai et al. found significant differences in the microbiomes when examining beta diversity, with the smallest clams clustering differently from the normal-sized and the largest clams in a PCA plot. Statistically significant clusters were also seen in NMDS plots of Bray-Curtis dissimilarity in Forberg et al.’s study on differently-sized juvenile killifish and cod larvae of the same age (2016).

Additionally, each spat size class had a distinct core microbiome as defined by Ainsworth et al. (2015). This definition of core microbiome was previously used in a study on oysters by Dubé et al. (2019). Dube et al. found that the core microbiome stabilized at the presence threshold of 70% of samples, while we observed thresholds of 52% and 56% for large and small spat, respectively. When not segregated by size, the core microbiome stabilized at the presence threshold of 61% of samples, indicating that segregation of the core microbiome by animal size was meaningful. Only one taxon, *Mycoplasma* sp. (ASV01), was present in all samples. Because the definition of the core microbiome differs by study (King et al., 2012; King et al., 2019a; King et al., 2019b; King et al., 2020), and researchers have examined the microbiomes of different compartments of the oyster separately, it is difficult to compare core microbiomes among studies.

Given that the gut microbial community of oysters is proposed to be involved with nutrient absorption (Kesarcodi-Watson et al., 2008), selecting particular residents would appear to benefit the host. To test this hypothesis, we explored potential functional contributions of the differentially abundant taxa between size classes in our study, focusing primarily on the members of the microbiome that increase in proportion in larger animals. We detected that 16 ASVs were statistically more abundant in the large spat. Most ASVs that were differentially abundant in the small oysters were very low in abundance, further supporting our conclusion that these taxa may be selectively removed from the microbiomes of large animals. The 16 ASVs differentially abundant in the large juveniles made up 53.6% of the reads in all samples, while the three ASVs differentially abundant in the small juveniles only made up 1.6% of all reads. It is possible that the three taxa that were differentially abundant in small oysters simply had not yet been passively lost in the development of the microbiome. Based on previously described phylogenetic-functional relationships, the ASVs differentially found in the large oysters appear to show or possess capabilities of the degradation of macromolecules, including hydrocarbons and sugars.

ASV01, *Mycoplasma* sp., made up 25.81% of ASVs in large samples on average. *Mycoplasma* has been reported to be consistently abundant in studies of the microbiome of *C. gigas*, dominating the gill (Wegner et al., 2013), gut (Lokmer et al., 2016; Arfken et al., 2021), stomach, (King et al., 2012), and digestive gland (King et al., 2020). *Mycoplasma* has been documented not only in bivalves, but also in other aquatic animals. Aronson et al. (2017) found that *Mycoplasma* species accounted for over half of the 16S rRNA reads in the digestive gland of deep-sea snail *Rubyspira osteovora*. Additionally, *Mycoplasma* spp. represented 90% of the reads from tissue homogenates of the marine mollusc *Elysia rufescens* (Davis et al., 2013).

Though no studies to date have shown the precise role of *Mycoplasma* species in their hosts, their consistent dominance among host microbiomes, combined with the fact that we observed the genus differentially abundant in large oysters during an early life stage, possibly suggests a role in digestion, specifically biomass conversion for animal growth. This phenomenon has been observed in Atlantic salmon as a positive correlation between relative *Mycoplasma* sp. abundance in the gut and animal weight (Bozzi et al., 2021). Fraune and Zimmer (2008) found a positive correlation between a *Mycoplasma*-like symbiont (Candidatus *Hepatoplasma crinochetorum*, within the order Mycoplasmatales) in the midgut glands of the terrestrial isopod host *Porcellio scaber* and the survival of the host on low-quality, cellulose-rich food. This bacterial taxon was the only one detected in isopods that survived. Furthermore, the presence of this taxon did not confer a benefit to the host when a high-quality, easily digested food source was offered instead. Their findings suggest that this *Mycoplasma*-like symbiont may aid in acquiring energy from refractory food sources. Wang et al. (2016) found multiple copies of genes involved in oligosaccharide degradation and proteolysis in two *Mycoplasma* species recovered from the stomachs of deep-sea isopods, indicating the potential to aid the host in breaking down and acquiring energy from food sources.

*Mycoplasma* spp. have also been implicated in marine animal health regarding disease state, though findings differ regarding positive or negative correlations with host disease status. Cleressi et al. (2020) noted that Mycoplasmataceae was more abundant in oysters with increased susceptibility to oyster herpesvirus type 1 (OsHV-1). Similarly, *Mycoplasma* was a predictor of mortality in oysters that succumbed to OsHV-1 in a study by Delisle et al. (2022). In contrast, Green and Barnes (2010) reported that the sister taxon to ASV01 was present in the digestive glands of Sydney rock oysters not infected with the parasite *Marteilia sydneyi*, but it was absent in infected samples. Related to this observation, a high abundance of *Mycoplasma* has been associated with better health with regard to disease status in Atlantic salmon (Bozzi et al., 2021).

Given that *Mycoplasma* spp. are often obligate parasites with reduced genomes and intracellular lifestyles, it is likely that their co-existence with a host is highly developed. Rasmussen et al. (2021) used metagenome assemblies of abundant *Mycoplasma* spp. associated with salmonid hosts and found genes specific to ammonia use in the biosynthesis of amino acids. They also found other biosynthetic genes encoded by *Mycoplasma* that complement gaps in the biosynthetic capabilities of their salmonid host, suggesting a mutualistic relationship between host and symbiont. Similar work has been carried out in oysters, possibly beginning to explain why *Mycoplasma* may be so abundant. Pimentel et al. (2021) performed a metagenome assembly analysis on multiple Mollicutes species (the family to which *Mycoplasma* belongs) abundant in eastern oysters and found reduced genomes that suggest a reliance upon the host for nutrients. Additionally, they found two energy acquisition pathways involving chitin and arginine deiminase that were unique only to the *Mycoplasma* species found in the eastern oyster gut and absent in closely related *Mycoplasma* spp., indicating a symbiotic relationship.

ASV10, Rhodospirillaceae, had the highest effect size of all differentially abundant taxa (see supplemental material) and constituted 5.56% of ASVs in large samples on average. A SINA search classified the taxa as *Marispirillum* sp. Many *Marispirillum* species have been associated with hydrocarbon and lipid degradation. A BLAST search (NCBI Resource Coordinators, 2018) found that the closest hit to ASV10 was *M. indicum*, a bacterium originally isolated from a crude oil-degrading microbial consortium in the Indian Ocean (Lai et al., 2009) and closely related to other *Marispirillum* spp. that have also been associated with oil wells and tarballs (Shinde et al., 2018). *M. indicum* was shown to emulsify lipids (Gomes et al., 2018). Given the phylogenetic relationships, the Rhodospirillaceae ASV10 may be involved with lipid degradation, which may provide a source of energy to their hosts. Further nutrients may be provided to their hosts through nitrogen cycling; a metagenomic study on sponge-associated Rhodospirillaceae found taxa capable of carrying out various parts of the denitrification process (Karimi et al., 2018). Interestingly, Cleressi et al. (2020) found that a higher abundance of Rhodospirillaceae was linked to susceptibility to OsHV-1; however, in another study, King et al. did not note this taxon as being related to OsHV-1 (2019a). Cleressi et al. noted two other taxa associated with increased mortality from OsHV-1, both of which (Vibrionaceae and Mycoplasmataceae) followed the same pattern in our data as Rhodospirillaceae, wherein they were more abundant in the large oysters.

ASV09 was assigned to the Vibrionaceae family and made up 4.37% of ASVs on average in large spat samples. Many members of the Vibrionaceae belong to the *Vibrio* genus, which are often pathogenic to oysters (Elston et al., 2008; Arunkumar et al., 2020). Wegner et al. (2019) found that the pathogenicity of *Vibrio* spp. to Pacific oyster larvae was dependent on whether the species was allopatric or sympatric, with sympatric species being less harmful to larvae. Furthermore, Kapareiko et al. (2011) discovered a *Vibrio* sp. with probiotic effects that acted antagonistically toward pathogenic *Vibrio* in eastern oysters (*Crassostrea virginica*). Madison et al. (2022) have also observed antipathogenic activity of potentially probiotic *Vibrio* sp. Thus, it is possible that ASV09 behaves more like a beneficial bacterium than a harmful one within the *C. gigas* microbiome. Dai et al. (2022) found contrasting results in their study examining differences in mean abundance among three different size classes of clams regarding the proportion of Vibrionaceae. In that study, Vibrionaceae was more abundant in the largest compared to the smallest size class, but it was also more abundant in the smallest compared to the medium size class. The authors proposed that because it was more abundant in the extreme size classes compared to the medium size class, the increased relative abundance of Vibrionaceae may indicate a disturbance to the digestive system related to abnormal growth.

ASV11, *Psychromonas* sp., made up an average of 3.75% of ASVs in large samples. SINA classified the sequence as *Psychromonas* sp. In the deep-sea bone-eating snail *Rubyspira osteovora*, *Psychromonas* was second only to *Mycoplasma* in gut microbiome abundance (Aronson et al., 2017). Similar to *Mycoplasma* spp., *Psychromonas* in symbiotic relationships with eukaryotic hosts have more reduced genomes compared to free-living *Psychromonas*, with the absence of certain metabolic genes indicating adaptation to a specific environment within the host (Zhang et al., 2018). In that study, the genome of the symbiotic *Psychromonas* was only about half the size of free-living species.

ASV13, Spirochaetaceae, made up an average of 2.95% of ASVs in large oyster samples. The SINA aligner classified ASV13 as belonging to the genus *Salinispira* within the family Spirochaetaceae. *S. pacifica* is an obligate fermenter of glucose discovered in a hypersaline mat (Ben Hania et al., 2015). Gao et al. (2022) found *Salinispira* in saline oil-contaminated soil. Currently, there is no literature exploring the functions of *Salinispira* in host microbiomes. King et al. (2020) found that Spirochaetaceae were the only taxon that were conserved across all *C. gigas* samples in six different estuaries.

ASV06, *Aliikangiella*, made up an average of 3.40% of ASVs in large samples. Literature on *Aliikangiela* is sparse at this time; however, Wang et al. (2018) performed genomic analysis on several strains of *Kangiella*, within the same family as *Aliikangiella*, and found reduced genome sizes in conjunction with many diverse genes relating to extracellular protein degradation. ASV20, *Colwellia*, made up 1.66% of all reads in large samples. *Colwellia* sp. was isolated from an oil spill plume and had genes for hydrocarbon degradation (Mason et al., 2014). ASV22, Devosiaceae, made up 1.43% of all reads in large samples. Devosiaceae are associated with marine biofilms on wood and plastics (Kesy et al., 2019) and have been found in hydrocarbon-rich environments, indicating a potential to degrade hydrocarbons (Kumar et al., 2008). Eight ASVs were differentially abundant in large oyster samples but had low abundance (they did not reach an average of ≥ 1% abundance in large samples). The proposed functions for all these taxa are summarized in **Table 1**.

**Table 1:** Proposed functional roles of ASVs differentially abundant in large spat, sorted by relative abundance.

Taxonomic ID	Mean relative abundance in large spat (% of reads)	Proposed functions	Source(s)
asv01 (*Mycoplasma*)	25.81	Biomass conversion/digestion	Fraune and Zimmer, 2008; Wang et al., 2016; Bozzi et al., 2021; Rasmussen et al., 2021
asv10 (Rhodospirillaceae)	5.56	Hydrocarbon degradation, lipid emulsion, nitrate reduction, ammonification	Lai et al., 2009; Gomes et al., 2018; Karimi et al., 2018; Shinde et al., 2018
asv09 (Vibrionaceae)	4.37	Antagonism towards pathogens	Kapareiko et al., 2011; Madison et al., 2022
asv11 (*Psychromonas*)	3.75	Unknown	
asv06 (*Aliikangiella*)	3.40	Extracellular protein degradation	Wang et al., 2018
asv13 (Spirochaetaceae)	2.95	Potential oil degradation	Gao et al., 2022
asv20 (*Colwellia*)	1.60	Hydrocarbon degradation	Mason et al., 2014
asv22 (Devosiaceae)	1.35	Potential hydrocarbon degradation	Kumar et al., 2008; Kesy et al., 2019
asv29 (*Maritalea*)	0.67	Nitrate reduction	Fukui et al., 2012; Zhukova et al., 2022
asv72 (Helicobacteraceae)	0.54	Sulfide oxidation	Murray et al., 2016; Nakagawa et al., 2017
asv41 (*Salinirepens*)	0.50	Sulfate reduction, organic matter decomposition	Espín et al., 2021
asv89 (Oligoflexaceae)	0.44	Nutrient turnover	Kieft et al., 2020
asv62 (*Lentilitoribacter*)	0.43	Unknown	
asv61 (*Halobacteriovorax*)	0.38	Nutrient cycling, anti-pathogen activity	Chen et al., 2018
asv48 (Rhizobiales)	0.35	Nitrogen fixation, aromatic compound degradation	Gomes et al., 2014; Liu et al., 2019
asv82 (*Terasakiella*)	0.29	Denitrification, inhibition of sulfate-reducing bacteria	Bødtker et al., 2009

While this study is robust in its 16S rRNA amplicon-based community analysis, there are limitations to this study and areas that can be further explored. PCR-based methods of community analysis, while providing impressive depth of sequencing per sample on the Illumina MiSeq, introduce many biases that can be avoided with PCR-independent methods (Bonk et al., 2018). Additionally, by only sequencing the V4 region of the 16S gene instead of the entire 1500bp gene, it is impossible to assign ASVs down to the taxonomic level of species (Johnson et al., 2019). Given that different species within the same genus can have variable metabolisms (Vieira-Silva et al., 2016), this discrepancy has important implications in the potential functional role of each ASV. Further explorations of the role of microbiome constituents may include metagenome sequencing, which provides a more accurate representation of the given community than 16S amplicon-based analysis (Jovel et al., 2016).

The observed compositional shift in beta diversity is due to differentially abundant taxa. While we cannot distinguish whether the change in the microbiome is a result of or cause of changes in body size, we propose that the specific changes in composition indicate that the more dominant taxa found in larger animals confer specific benefits upon animal performance. We suspect that the physiological capabilities of the specific microbiome members that change among animal size is related to animal growth and performance, although controlled experiments and full (meta)genome sequencing is necessary to fully support the proposed hypotheses. These findings can have significant implications on the mechanisms influencing the host-microbiome relationship to nutrition and the discovery of new probiotics, which can be employed in aquaculture systems to help maximize production yields.

## Data availability statement

The datasets presented in this study can be found in online repositories. The names of the repository/repositories and accession number(s) can be found below: https://www.ncbi.nlm.nih.gov/bioproject/PRJNA886437/, PRJNA886437.

## Author contributions

RM, CL, and CS acquired funding for the project. CL conceived of the study idea. ME contributed to sample processing. ME and RM analyzed data. ME wrote the manuscript. All authors contributed to the article and approved the submitted version.
