# Supplementary material for: Dominant bacterial taxa drive microbiome differences of juvenile Pacific oysters of the same age and variable sizes
Source: Front Microbiomes. 2023 Mar 30;2:1071186. doi: 10.3389/frmbi.2023.1071186 (PMC12993558; doi:10.3389/frmbi.2023.1071186)
Supplement: Supplementary file 1 [file DataSheet_1.docx]

Supplementary Material


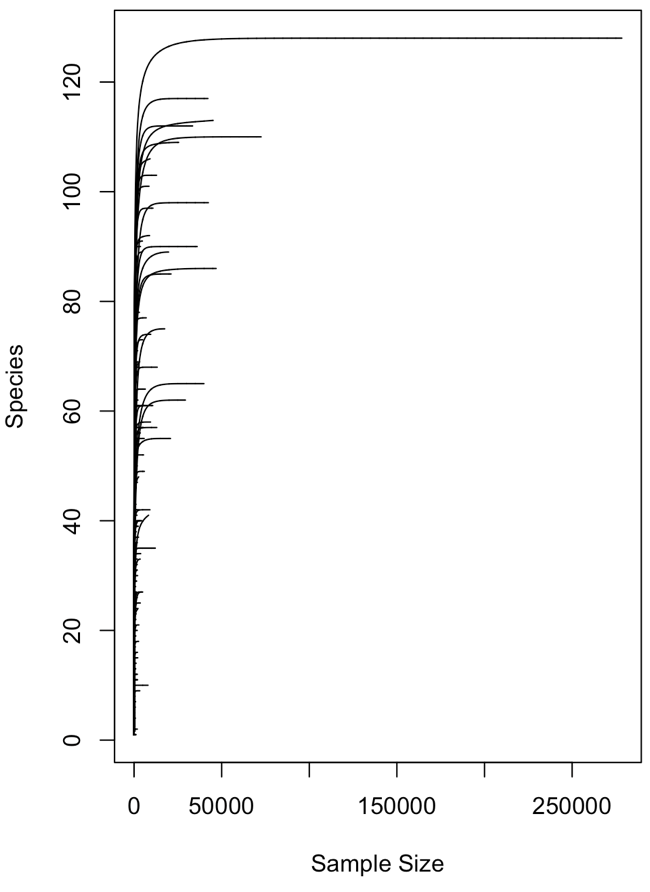

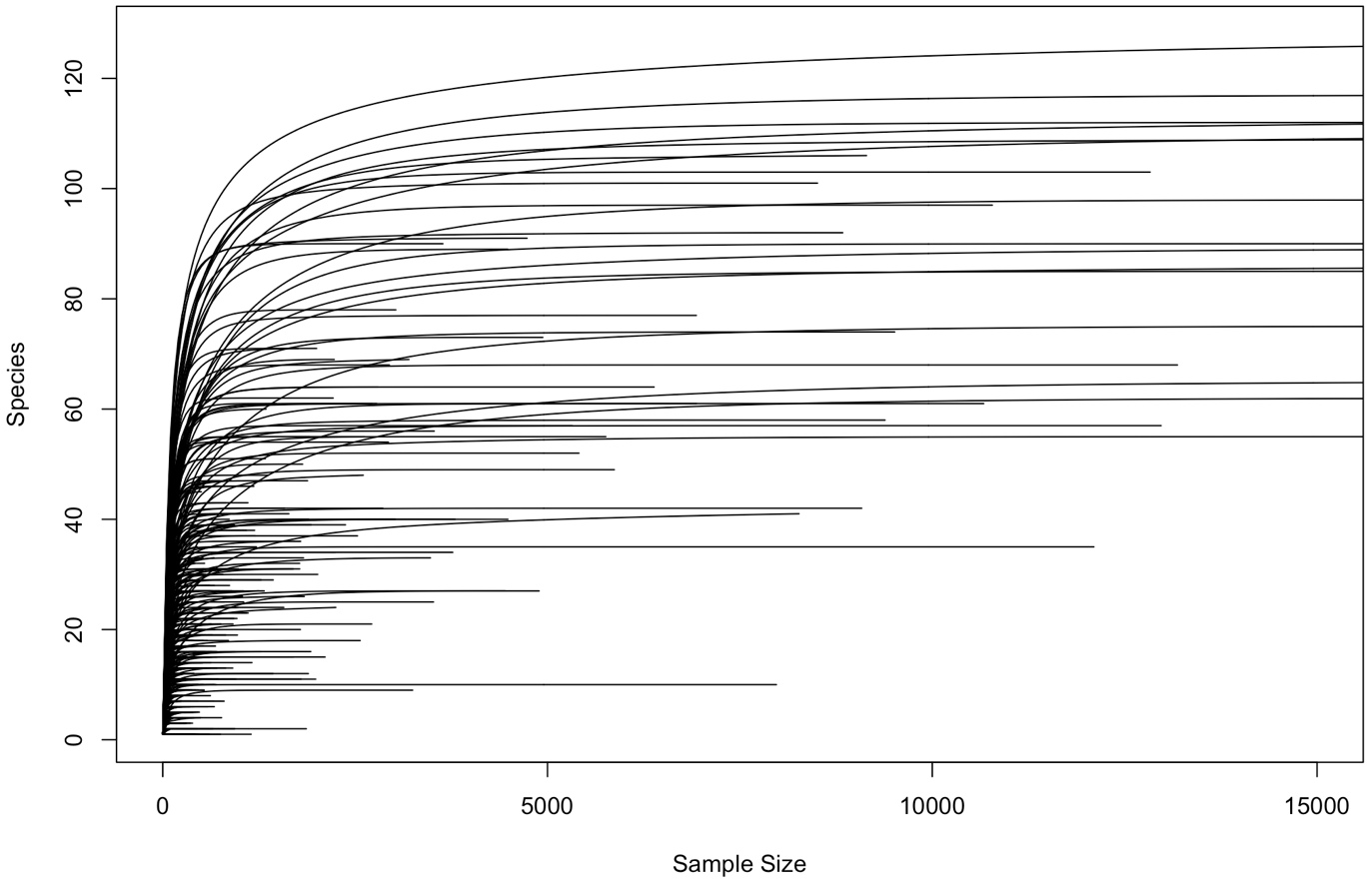


**Figure S1**. Rarefaction curve for the original 147 libraries (left), zoomed in to focus on the region between 5000 and 15000 reads (right). The number of unique species in each sample consistently began to level out around 5000 reads and close to fully leveled out at 15000, so we chose these numbers for read depth limits.


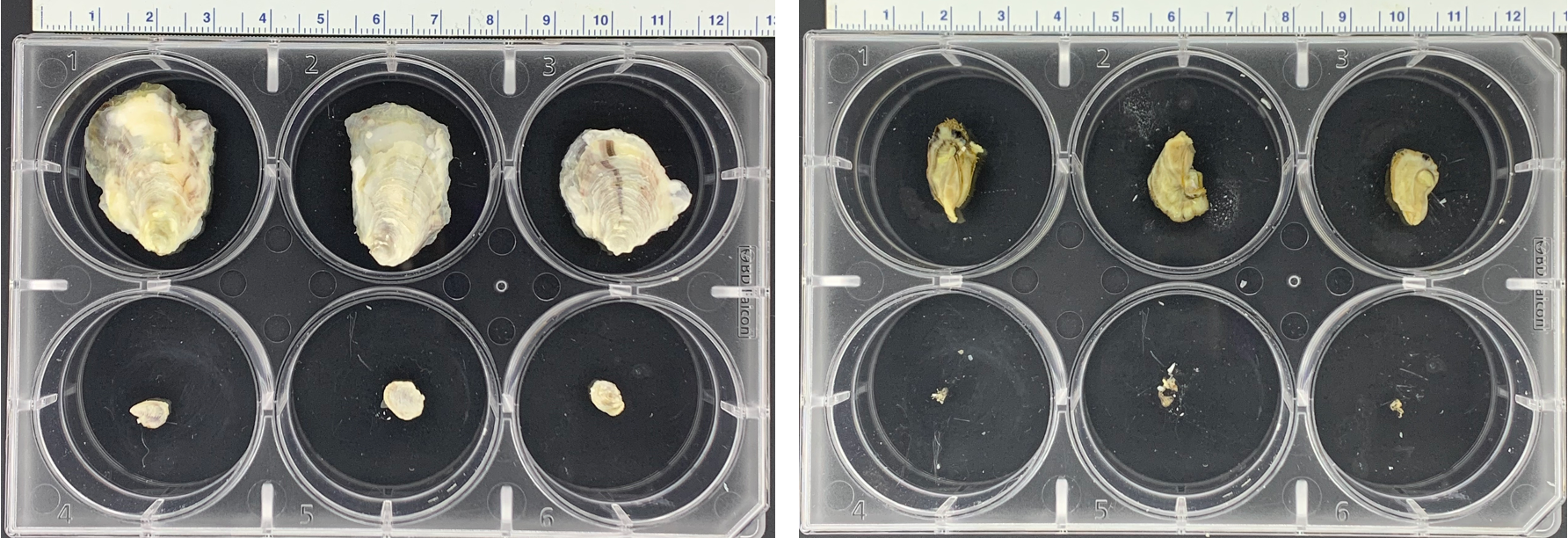


**Figure S2**. Representative large (top row) and small (bottom row) spat, unshucked (left panel) and shucked (right panel). Ruler is in cm.


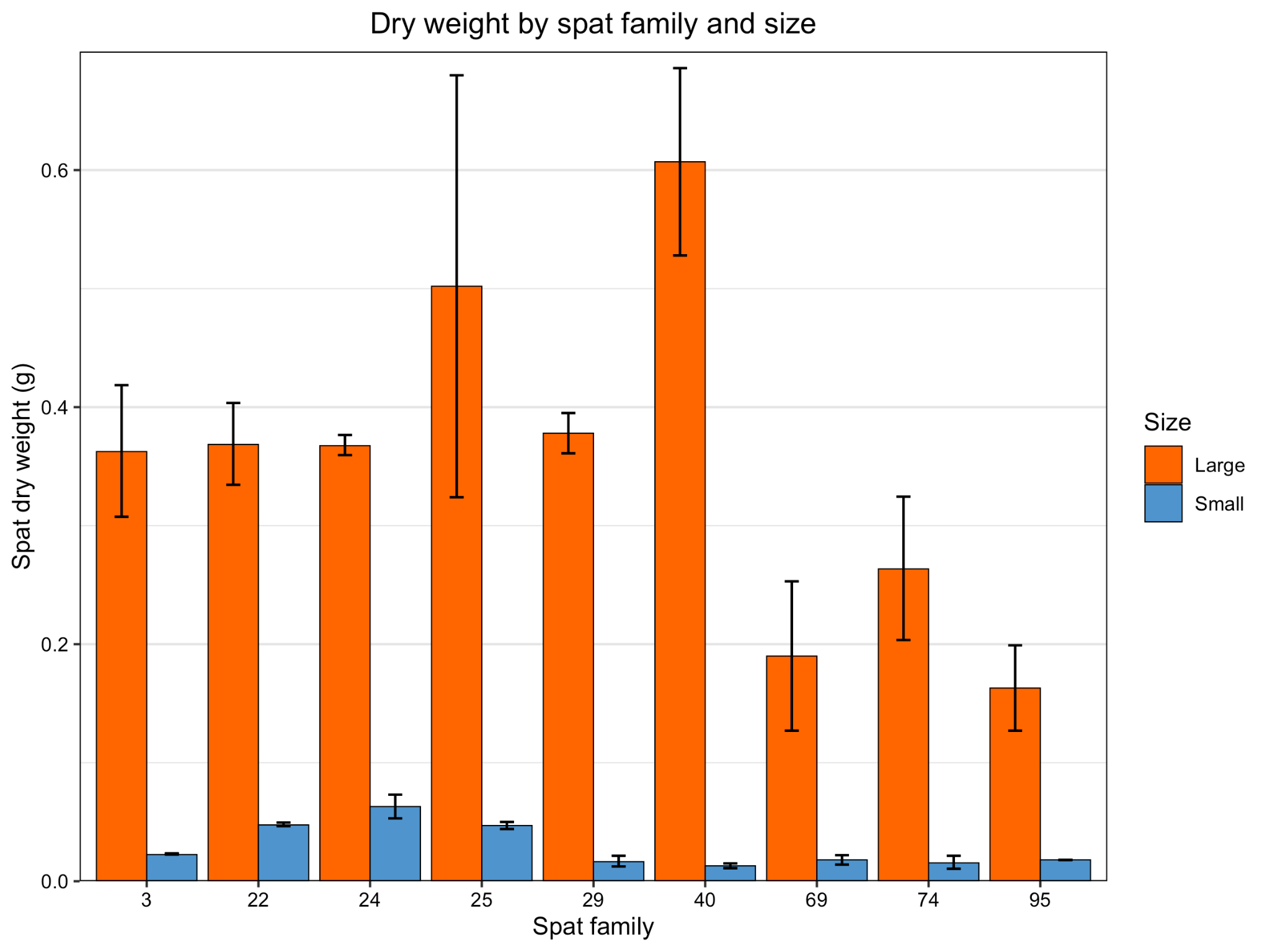


**Figure S3**. Spat dry weight by family. Measurements include both dry tissue and shell. Dataset was too small (n = 2 per size class per family) to statistically validate between-family differences. Error bars represent standard deviation of the two samples.


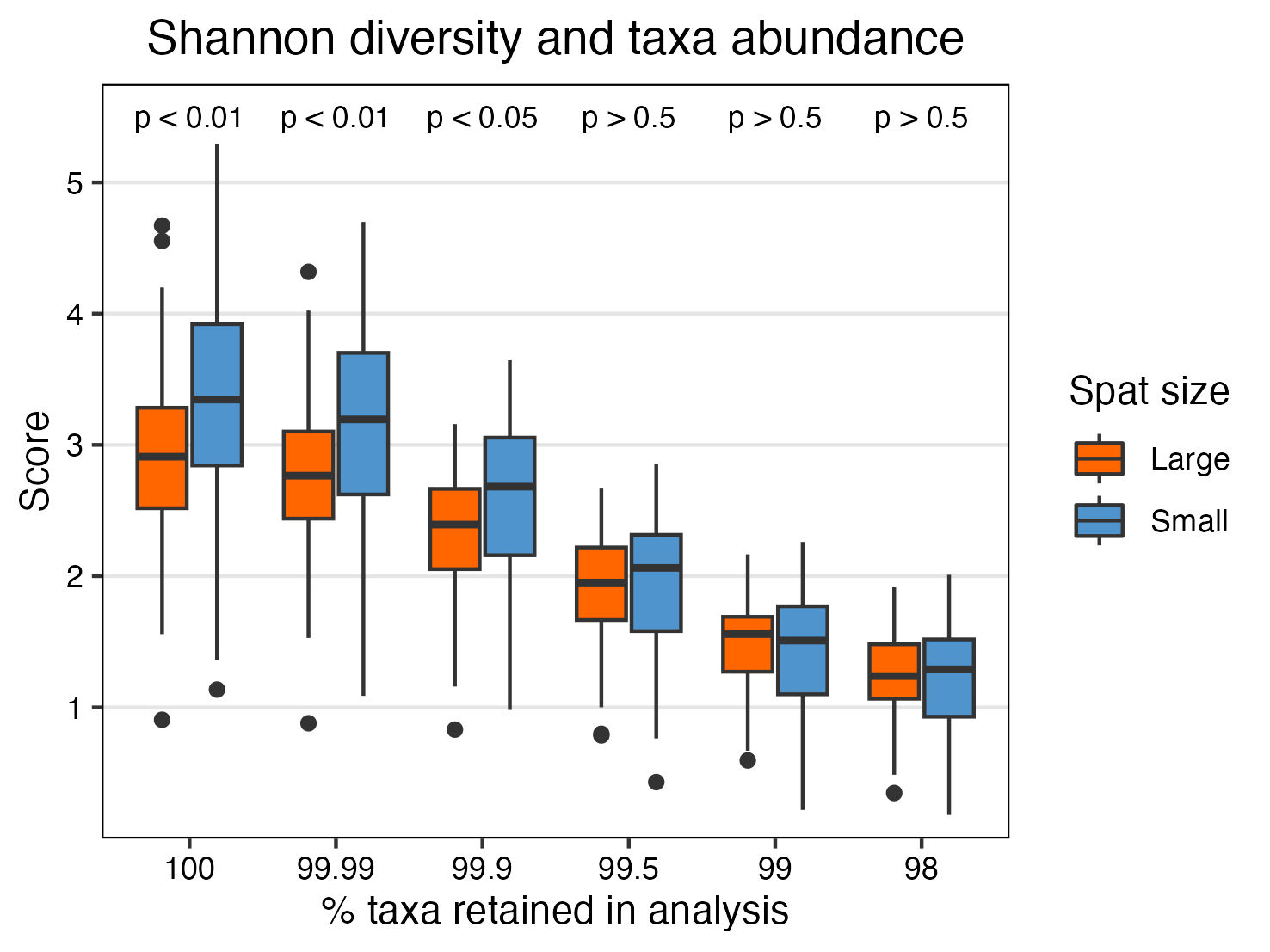


**Figure S4**. Shannon alpha diversity measures on the data with an increasing number of rare taxa removed (i.e., 99.99 % taxa retention contains taxa accounting for > 99.99% of all reads; 99.9% retains taxa that accounted for > 99.9% of all reads, etc.). The statistical difference in scores between size classes (p-values) ceases at 99.5% taxa retention. The purpose of this figure is to provide evidence for rare taxa driving the differences seen in diversity measures that in incorporate evenness (Fig. 2). The scores shown here are different from in Fig. 2 because the data was not normalized in PhyloseqCompanion, as we did not wish to compare scores among metrics.


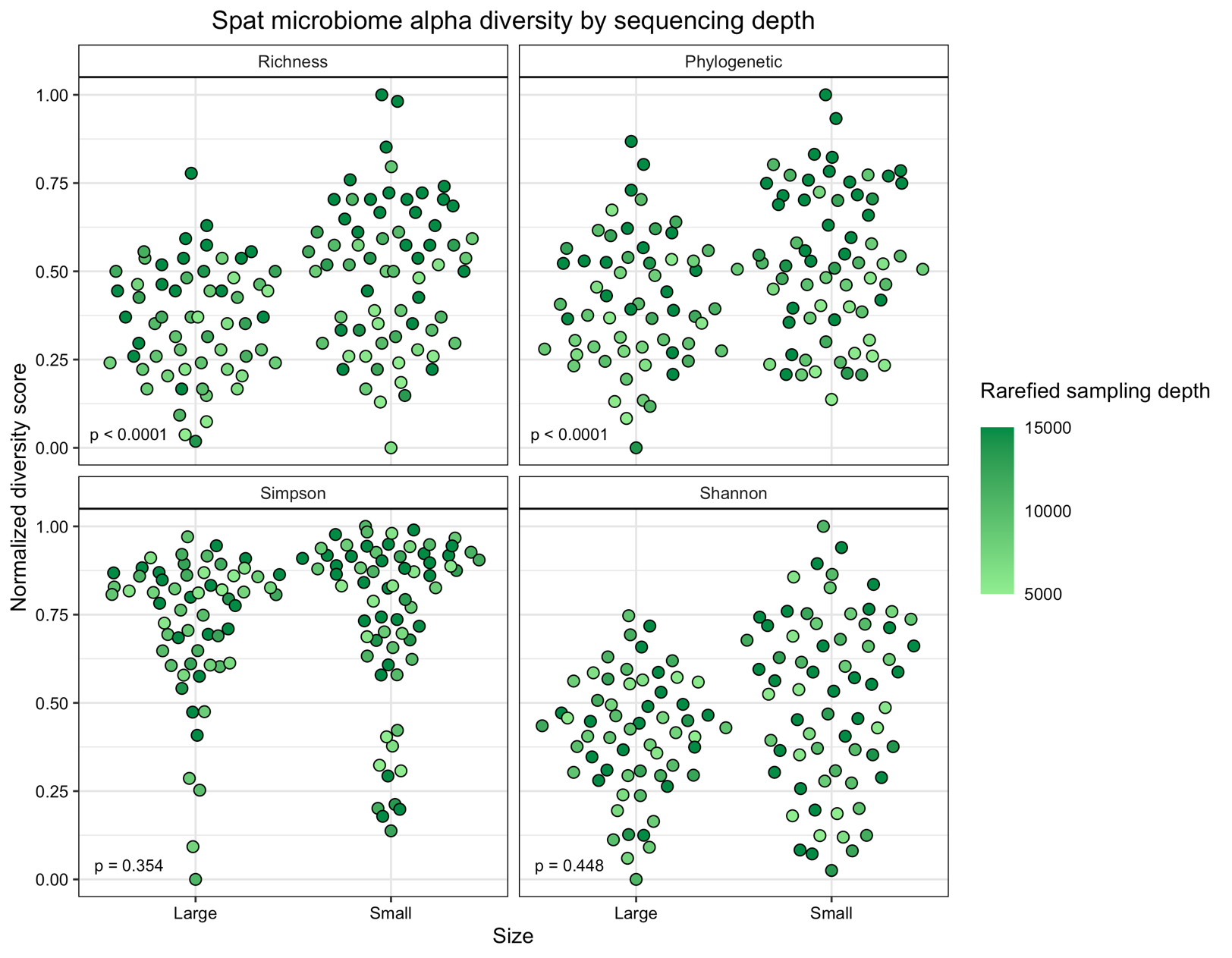


**Figure S5**. Normalized alpha diversity score of four different metrics (clockwise from top left: richness, Faith’s phylogenetic distance, Shannon index, and Simpson index). In richness and phylogenetic measures of alpha diversity, slight but statistically significant trends can be seen in which samples with higher rarefied read depth (dark green) show higher alpha diversity than samples with lower rarefied sampling depth (light green).


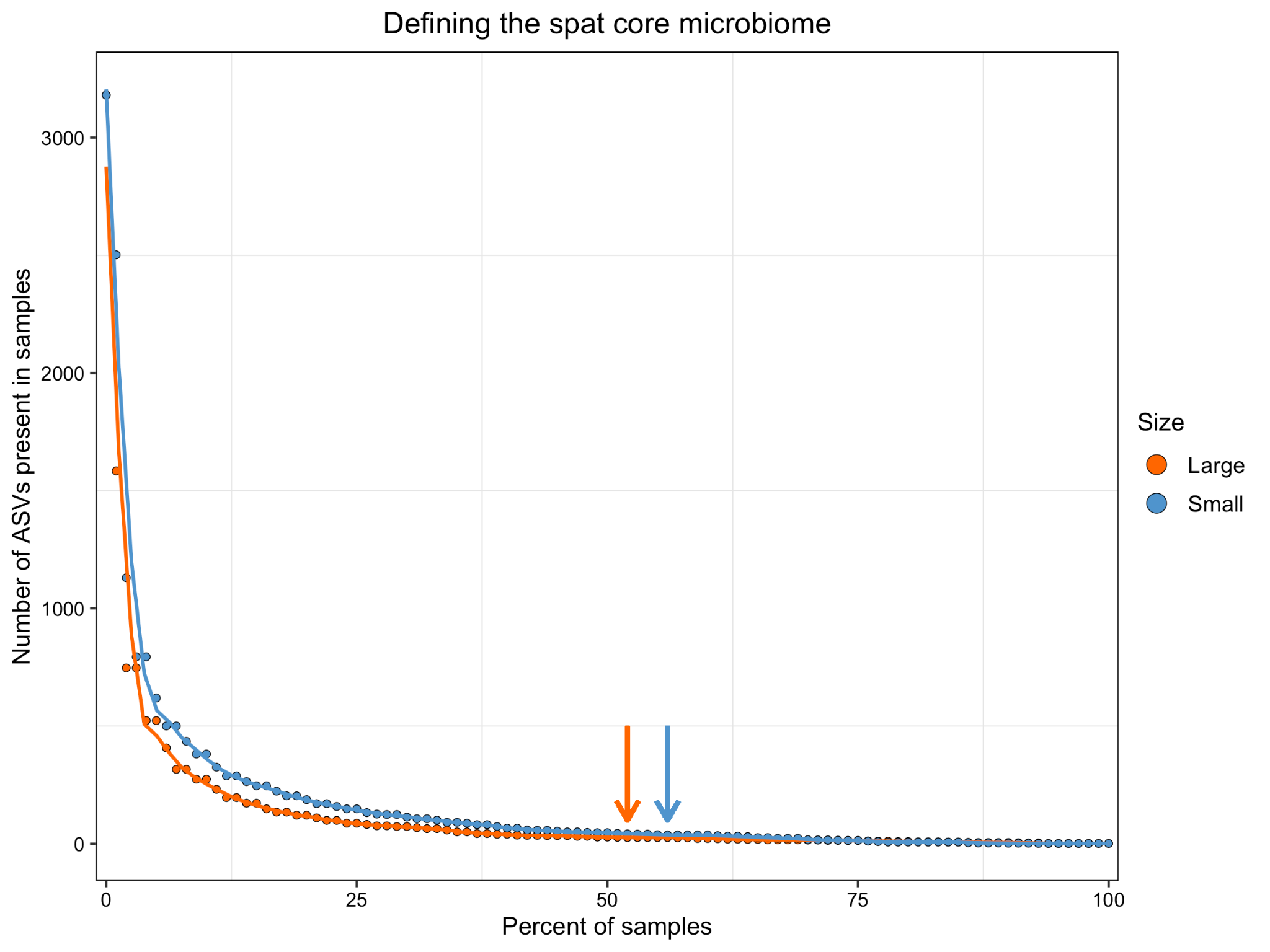


**Figure S6**. The core microbiome for each size was defined by the ASVs present at a stabilized number of samples. This corresponded to 26 ASVS that were present in 52% of large samples (orange arrow) and 35 ASVS that were present in 56% of small samples (blue arrow). The number of samples in a size class was increased sequentially by 1%, and the percentage of samples at which the number of ASVs stabilized over a 4% increase in samples was defined as the core microbiome of that size class. Individual points represent numbers of ASVs at a given percentage of samples in a size class.

**Table S1**. Tracking file of raw reads through filter steps in the DADA2 pipeline from raw to rarefied reads. Explanations of each column can be found in the DADA2 literature [(Callahan et al., 2016)](https://www.zotero.org/google-docs/?dMCdu4) and corresponding tutorial.

| **Sample** | **input** | **filtered** | **Denoised (fwd)** | **Denoised (rev)** | **merged** | **nonchimeric** | **rarefied** |
| --- | --- | --- | --- | --- | --- | --- | --- |
| 22L1 | 47913 | 39955 | 39219 | 39201 | 36713 | 35900 | 15000 |
| 22L2 | 34532 | 29853 | 29193 | 29283 | 28684 | 27458 | 15000 |
| 22L3 | 29204 | 25726 | 25250 | 25223 | 24678 | 23724 | 15000 |
| 22L4 | 23081 | 19474 | 18763 | 18786 | 17277 | 16965 | 15000 |
| 22L5 | 5775 | 4180 | 3933 | 3884 | 3716 | 3666 | NA |
| 22L6 | 19554 | 14481 | 13617 | 13461 | 12705 | 12343 | 12343 |
| 22L7 | 43449 | 32390 | 31711 | 31684 | 30190 | 29832 | 15000 |
| 22L8 | 16177 | 13562 | 13192 | 13179 | 12607 | 12543 | 12543 |
| 22S1 | 17289 | 13302 | 12350 | 12469 | 11163 | 10893 | 10893 |
| 22S2 | 24740 | 19667 | 19133 | 19096 | 18303 | 17652 | 15000 |
| 22S3 | 76754 | 59808 | 58758 | 58829 | 55084 | 53723 | 15000 |
| 22S4 | 19558 | 15564 | 15010 | 15045 | 14382 | 14113 | 14113 |
| 22S6 | 22181 | 16839 | 15983 | 15915 | 14750 | 13863 | 13863 |
| 22S7 | 32071 | 25394 | 24313 | 24451 | 23153 | 22730 | 15000 |
| 22S8 | 32024 | 25092 | 24505 | 24497 | 22367 | 21748 | 15000 |
| 24L1 | 10749 | 8274 | 7466 | 7483 | 6776 | 4765 | NA |
| 24L2 | 14404 | 11806 | 11014 | 11154 | 10554 | 9646 | 9646 |
| 24L3 | 26879 | 22249 | 21299 | 21389 | 20669 | 18956 | 15000 |
| 24L4 | 17700 | 14490 | 13893 | 13975 | 13546 | 11863 | 11863 |
| 24L5 | 23319 | 17821 | 17005 | 16960 | 15862 | 15403 | 15000 |
| 24L6 | 18688 | 14307 | 13317 | 13402 | 12595 | 10165 | 10165 |
| 24L7 | 13547 | 9208 | 8400 | 8396 | 7931 | 3552 | NA |
| 24L8 | 14096 | 10580 | 9832 | 9794 | 9117 | 7518 | 7518 |
| 24S1 | 48132 | 14411 | 13896 | 13458 | 12504 | 12302 | 12302 |
| 24S2 | 22250 | 17148 | 16159 | 16046 | 14543 | 10754 | 10754 |
| 24S3 | 16079 | 13417 | 12560 | 12614 | 11676 | 11630 | 11630 |
| 24S4 | 14296 | 11630 | 10788 | 10923 | 10159 | 9939 | 9939 |
| 24S5 | 97264 | 77512 | 75569 | 75967 | 71390 | 64750 | 15000 |
| 24S6 | 10050 | 8106 | 7091 | 7176 | 6023 | 5797 | 5797 |
| 24S7 | 6448 | 5238 | 4514 | 4490 | 3803 | 3750 | NA |
| 24S8 | 4731 | 3681 | 3259 | 3205 | 2869 | 2655 | NA |
| 25L1 | 14533 | 11687 | 11150 | 11163 | 10487 | 10113 | 10113 |
| 25L2 | 12820 | 10569 | 10286 | 10321 | 10184 | 9106 | 9106 |
| 25L3 | 18041 | 15338 | 14999 | 15077 | 14559 | 13826 | 13826 |
| 25L5 | 7864 | 3304 | 2743 | 2500 | 2094 | 1921 | NA |
| 25L6 | 12399 | 10177 | 9548 | 9546 | 9012 | 8546 | 8546 |
| 25L7 | 11316 | 9348 | 8081 | 8144 | 6605 | 6263 | 6263 |
| 25S1 | 18992 | 15161 | 14567 | 14584 | 13787 | 13403 | 13403 |
| 25 | 29322 | 25143 | 24427 | 24591 | 23586 | 22649 | 15000 |
| 25S3 | 42071 | 35166 | 33748 | 33969 | 31535 | 31109 | 15000 |
| 25S4 | 70 | 41 | 14 | 14 | 14 | 14 | NA |
| 25S5 | 4922 | 3624 | 3388 | 3361 | 3258 | 3103 | NA |
| 25S6 | 15237 | 11533 | 11076 | 10974 | 10237 | 9562 | 9562 |
| 25S7 | 11426 | 9192 | 8308 | 8281 | 7348 | 7255 | 7255 |
| 25S8 | 779 | 553 | 489 | 445 | 406 | 396 | NA |
| 29L1 | 4175 | 2850 | 2619 | 2508 | 2176 | 2158 | NA |
| 29L2 | 51432 | 40245 | 38813 | 38686 | 36465 | 35129 | 15000 |
| 29L3 | 11711 | 9834 | 9154 | 9134 | 8646 | 8255 | 8255 |
| 29L4 | 16534 | 12485 | 11779 | 11703 | 10145 | 9926 | 9926 |
| 29L5 | 26049 | 20888 | 20020 | 20003 | 19234 | 18114 | 15000 |
| 29L6 | 33576 | 28454 | 27587 | 27503 | 26144 | 25452 | 15000 |
| 29L7 | 16602 | 13364 | 12554 | 12602 | 11626 | 11192 | 11192 |
| 29L8 | 11749 | 9487 | 8867 | 8870 | 8151 | 7510 | 7510 |
| 29S1 | 17085 | 12821 | 12147 | 12108 | 10811 | 10794 | 10794 |
| 29S2 | 58301 | 45240 | 44175 | 44090 | 41515 | 41393 | 15000 |
| 29S3 | 73075 | 61038 | 60304 | 60222 | 58645 | 58186 | 15000 |
| 29S4 | 70804 | 54322 | 53030 | 53124 | 47107 | 46932 | 15000 |
| 29S5 | 28 | 12 | 1 | 1 | 0 | 0 | NA |
| 29S6 | 18302 | 14471 | 13633 | 13598 | 12450 | 12301 | 12301 |
| 29S7 | 25875 | 20445 | 19395 | 19356 | 17363 | 17266 | 15000 |
| 29S8 | 13101 | 8731 | 8179 | 8131 | 7330 | 6904 | 6904 |
| 40L1 | 57 | 32 | 3 | 3 | 0 | 0 | NA |
| 40L2 | 40636 | 34808 | 34323 | 34361 | 33619 | 33204 | 15000 |
| 40L3 | 19129 | 15435 | 14282 | 14329 | 12949 | 11552 | 11552 |
| 40L4 | 13412 | 10798 | 9697 | 9920 | 8819 | 7768 | 7768 |
| 40L5 | 13940 | 10297 | 9439 | 9413 | 8408 | 8083 | 8083 |
| 40L6 | 12068 | 9302 | 8643 | 8676 | 8285 | 7668 | 7668 |
| 40L7 | 27403 | 22481 | 21431 | 21453 | 20299 | 19923 | 15000 |
| 40L8 | 14318 | 11341 | 10559 | 10588 | 9745 | 9210 | 9210 |
| 40S1 | 11428 | 9315 | 8908 | 8981 | 8575 | 8511 | 8511 |
| 40S2 | 22084 | 16802 | 16143 | 16164 | 15362 | 15039 | 15000 |
| 40S3 | 10787 | 8476 | 7600 | 7634 | 6842 | 6071 | 6071 |
| 40S4 | 30887 | 23110 | 22148 | 22212 | 21029 | 20582 | 15000 |
| 40S5 | 24241 | 17938 | 17238 | 17247 | 15290 | 14853 | 14853 |
| 40S6 | 14435 | 10559 | 9846 | 9884 | 8915 | 8474 | 8474 |
| 40S7 | 15578 | 12255 | 11360 | 11362 | 10225 | 10032 | 10032 |
| 40S8 | 10619 | 7890 | 6989 | 6975 | 6268 | 5656 | 5656 |
| 69L1 | 8145 | 6298 | 5930 | 5960 | 5784 | 5456 | 5456 |
| 69L2 | 12790 | 9652 | 9270 | 9293 | 9115 | 7383 | 7383 |
| 69L3 | 17379 | 13802 | 13028 | 13067 | 12269 | 11341 | 11341 |
| 69L4 | 28483 | 22223 | 21210 | 21186 | 19263 | 18725 | 15000 |
| 69L5 | 15577 | 4048 | 3830 | 3655 | 3340 | 3164 | NA |
| 69L6 | 855 | 666 | 490 | 471 | 428 | 408 | NA |
| 69L8 | 4 | 2 | 1 | 1 | 0 | 0 | NA |
| 69S1 | 19359 | 14940 | 14285 | 14217 | 13495 | 13144 | 13144 |
| 69S2 | 12069 | 9764 | 9161 | 9103 | 8619 | 8374 | 8374 |
| 69S3 | 14853 | 11900 | 10750 | 10790 | 9021 | 8659 | 8659 |
| 69S4 | 40064 | 31136 | 29752 | 29922 | 27120 | 26411 | 15000 |
| 69S5 | 18933 | 14135 | 13193 | 13204 | 11668 | 11507 | 11507 |
| 69S6 | 72808 | 57577 | 56625 | 56597 | 54531 | 53370 | 15000 |
| 69S7 | 19529 | 15701 | 14717 | 14715 | 13490 | 12915 | 12915 |
| 69S8 | 7058 | 5297 | 4890 | 4928 | 4419 | 4002 | NA |
| 74L1 | 7921 | 286 | 248 | 188 | 120 | 84 | NA |
| 74L2 | 24767 | 18291 | 17839 | 17771 | 17035 | 15226 | 15000 |
| 74L3 | 30711 | 22319 | 21745 | 21635 | 20661 | 20430 | 15000 |
| 74L4 | 20313 | 16060 | 15371 | 15274 | 14287 | 13873 | 13873 |
| 74L5 | 11615 | 8418 | 7932 | 7844 | 7347 | 6990 | 6990 |
| 74L6 | 11642 | 8939 | 8637 | 8641 | 8393 | 8358 | 8358 |
| 74S1 | 25124 | 19085 | 18587 | 18607 | 15614 | 15596 | 15000 |
| 74S2 | 26187 | 19430 | 19041 | 19010 | 17835 | 17352 | 15000 |
| 74S3 | 15217 | 12570 | 11933 | 11895 | 10616 | 10498 | 10498 |
| 74S4 | 13336 | 10642 | 9625 | 9733 | 8413 | 8114 | 8114 |
| 74S5 | 32412 | 7498 | 6952 | 6401 | 5031 | 4987 | NA |
| 74S6 | 15271 | 11834 | 11241 | 11225 | 10293 | 10051 | 10051 |
| 74S7 | 8672 | 6497 | 5873 | 5703 | 5174 | 5089 | 5089 |
| 74S8 | 13090 | 10339 | 9800 | 9841 | 9155 | 8883 | 8883 |
| 3L1 | 8506 | 6533 | 5928 | 5920 | 5482 | 5416 | 5416 |
| 3L2 | 8371 | 7000 | 6681 | 6655 | 6461 | 6340 | 6340 |
| 3L3 | 11401 | 8988 | 8582 | 8546 | 8250 | 8212 | 8212 |
| 3L4 | 19145 | 16449 | 15634 | 15603 | 14952 | 14883 | 14883 |
| 3L5 | 9361 | 7460 | 6738 | 6708 | 5871 | 5828 | 5828 |
| 3L6 | 7356 | 6013 | 5634 | 5639 | 5318 | 5113 | 5113 |
| 3L7 | 16378 | 13053 | 11955 | 12003 | 10119 | 9720 | 9720 |
| 3L8 | 10493 | 8034 | 7513 | 7447 | 7038 | 6757 | 6757 |
| 3S1 | 26225 | 21394 | 20407 | 20435 | 18836 | 18555 | 15000 |
| 3S2 | 46719 | 37684 | 36422 | 36386 | 34606 | 34289 | 15000 |
| 3S3 | 8115 | 6403 | 5704 | 5676 | 5079 | 5060 | 5060 |
| 3S4 | 14342 | 11276 | 10297 | 10228 | 9210 | 9177 | 9177 |
| 3S5 | 26140 | 20414 | 19317 | 19209 | 18044 | 17930 | 15000 |
| 3S6 | 29177 | 24254 | 23635 | 23592 | 23067 | 22971 | 15000 |
| 3S7 | 7106 | 5393 | 4758 | 4728 | 4224 | 4129 | NA |
| 3S8 | 13420 | 8223 | 7652 | 7705 | 6630 | 6595 | 6595 |
| 94L1 | 4493 | 3357 | 2904 | 2845 | 2551 | 2070 | NA |
| 94L2 | 9573 | 7445 | 7021 | 6966 | 6132 | 5479 | 5479 |
| 94L3 | 4900 | 3923 | 3680 | 3691 | 3603 | 3542 | NA |
| 94L4 | 4058 | 2945 | 2721 | 2653 | 2423 | 2183 | NA |
| 94L5 | 18821 | 14745 | 14097 | 13974 | 12474 | 12399 | 12399 |
| 94L6 | 13872 | 11492 | 10630 | 10633 | 9350 | 9164 | 9164 |
| 94L7 | 17242 | 13227 | 12978 | 12954 | 10571 | 10548 | 10548 |
| 94L8 | 15304 | 12732 | 11915 | 11970 | 10920 | 10862 | 10862 |
| 94S1 | 7465 | 6218 | 5793 | 5879 | 5621 | 5492 | 5492 |
| 94S2 | 12584 | 10558 | 10077 | 10113 | 9474 | 9345 | 9345 |
| 94S3 | 7190 | 5996 | 5593 | 5553 | 5167 | 5139 | 5139 |
| 94S4 | 10995 | 9524 | 8959 | 8941 | 8404 | 8199 | 8199 |
| 94S5 | 35685 | 29151 | 28405 | 28497 | 27104 | 26237 | 15000 |
| 94S6 | 7444 | 6545 | 6100 | 6130 | 5778 | 5443 | 5443 |
| 94S7 | 35310 | 28941 | 28287 | 28298 | 26649 | 26444 | 15000 |
| 94S8 | 24340 | 19746 | 19114 | 19107 | 15992 | 15924 | 15000 |
| 95L1 | 6333 | 1521 | 1385 | 1331 | 1254 | 1235 | NA |
| 95L2 | 16618 | 12238 | 11716 | 11709 | 9786 | 9688 | 9688 |
| 95L3 | 9673 | 7349 | 6729 | 6696 | 6193 | 5638 | 5638 |
| 95L4 | 13569 | 10435 | 9887 | 9983 | 9580 | 9095 | 9095 |
| 95L5 | 7668 | 5250 | 4968 | 4837 | 4682 | 4283 | NA |
| 95L6 | 12953 | 10306 | 9847 | 9723 | 9344 | 9024 | 9024 |
| 95L7 | 24971 | 19570 | 18867 | 18869 | 17645 | 17093 | 15000 |
| 95L8 | 15254 | 12501 | 12108 | 12125 | 11754 | 11650 | 11650 |
| 95S1 | 7735 | 6364 | 5944 | 5956 | 5624 | 5501 | 5501 |
| 95S2 | 36393 | 31370 | 30503 | 30569 | 29684 | 29086 | 15000 |
| 95S3 | 6780 | 5716 | 5338 | 5347 | 4925 | 4880 | NA |
| 95S4 | 25956 | 20905 | 20198 | 20270 | 18665 | 18548 | 15000 |
| 95S5 | 12326 | 4523 | 4120 | 3827 | 3266 | 3188 | NA |
| 95S6 | 9168 | 7500 | 6712 | 6820 | 6047 | 5763 | 5763 |
| 95S7 | 12467 | 9958 | 9238 | 9284 | 8283 | 8228 | 8228 |
| 95S8 | 14840 | 12102 | 11482 | 11479 | 10406 | 10047 | 10047 |

**Table S2.** Metadata corresponding to samples.

| **Sample** | **Spat family** | **Replicate** | **Size** | **Avg. wet weight (g)** | **Avg. dry weight (g)** |
| --- | --- | --- | --- | --- | --- |
| 22L1 | 22 | 1 | Large | 0.634 | 0.369 |
| 22L2 | 22 | 2 | Large | 0.634 | 0.369 |
| 22L3 | 22 | 3 | Large | 0.634 | 0.369 |
| 22L4 | 22 | 4 | Large | 0.634 | 0.369 |
| 22L6 | 22 | 6 | Large | 0.634 | 0.369 |
| 22L7 | 22 | 7 | Large | 0.634 | 0.369 |
| 22L8 | 22 | 8 | Large | 0.634 | 0.369 |
| 24L2 | 24 | 2 | Large | 0.639 | 0.368 |
| 24L3 | 24 | 3 | Large | 0.639 | 0.368 |
| 24L4 | 24 | 4 | Large | 0.639 | 0.368 |
| 24L5 | 24 | 5 | Large | 0.639 | 0.368 |
| 24L6 | 24 | 6 | Large | 0.639 | 0.368 |
| 24L8 | 24 | 8 | Large | 0.639 | 0.368 |
| 25L1 | 25 | 1 | Large | 0.985 | 0.502 |
| 25L2 | 25 | 2 | Large | 0.985 | 0.502 |
| 25L3 | 25 | 3 | Large | 0.985 | 0.502 |
| 25L6 | 25 | 6 | Large | 0.985 | 0.502 |
| 25L7 | 25 | 7 | Large | 0.985 | 0.502 |
| 29L2 | 29 | 2 | Large | 0.693 | 0.378 |
| 29L3 | 29 | 3 | Large | 0.693 | 0.378 |
| 29L4 | 29 | 4 | Large | 0.693 | 0.378 |
| 29L5 | 29 | 5 | Large | 0.693 | 0.378 |
| 29L6 | 29 | 6 | Large | 0.693 | 0.378 |
| 29L7 | 29 | 7 | Large | 0.693 | 0.378 |
| 29L8 | 29 | 8 | Large | 0.693 | 0.378 |
| 3L1 | 3 | 1 | Large | 0.635 | 0.363 |
| 3L2 | 3 | 2 | Large | 0.635 | 0.363 |
| 3L3 | 3 | 3 | Large | 0.635 | 0.363 |
| 3L4 | 3 | 4 | Large | 0.635 | 0.363 |
| 3L5 | 3 | 5 | Large | 0.635 | 0.363 |
| 3L6 | 3 | 6 | Large | 0.635 | 0.363 |
| 3L7 | 3 | 7 | Large | 0.635 | 0.363 |
| 3L8 | 3 | 8 | Large | 0.635 | 0.363 |
| 40L2 | 40 | 2 | Large | 0.935 | 0.607 |
| 40L3 | 40 | 3 | Large | 0.935 | 0.607 |
| 40L4 | 40 | 4 | Large | 0.935 | 0.607 |
| 40L5 | 40 | 5 | Large | 0.935 | 0.607 |
| 40L6 | 40 | 6 | Large | 0.935 | 0.607 |
| 40L7 | 40 | 7 | Large | 0.935 | 0.607 |
| 40L8 | 40 | 8 | Large | 0.935 | 0.607 |
| 69L1 | 69 | 1 | Large | 0.295 | 0.19 |
| 69L2 | 69 | 2 | Large | 0.295 | 0.19 |
| 69L3 | 69 | 3 | Large | 0.295 | 0.19 |
| 69L4 | 69 | 4 | Large | 0.295 | 0.19 |
| 74L2 | 74 | 2 | Large | 0.439 | 0.264 |
| 74L3 | 74 | 3 | Large | 0.439 | 0.264 |
| 74L4 | 74 | 4 | Large | 0.439 | 0.264 |
| 74L5 | 74 | 5 | Large | 0.439 | 0.264 |
| 74L6 | 74 | 6 | Large | 0.439 | 0.264 |
| 94L2 | 94 | 2 | Large | NA | NA |
| 94L5 | 94 | 5 | Large | NA | NA |
| 94L6 | 94 | 6 | Large | NA | NA |
| 94L7 | 94 | 7 | Large | NA | NA |
| 94L8 | 94 | 8 | Large | NA | NA |
| 95L2 | 95 | 2 | Large | 0.276 | 0.163 |
| 95L3 | 95 | 3 | Large | 0.276 | 0.163 |
| 95L4 | 95 | 4 | Large | 0.276 | 0.163 |
| 95L6 | 95 | 6 | Large | 0.276 | 0.163 |
| 95L7 | 95 | 7 | Large | 0.276 | 0.163 |
| 95L8 | 95 | 8 | Large | 0.276 | 0.163 |
| 22S1 | 22 | 1 | Small | 0.075 | 0.048 |
| 22S2 | 22 | 2 | Small | 0.075 | 0.048 |
| 22S3 | 22 | 3 | Small | 0.075 | 0.048 |
| 22S4 | 22 | 4 | Small | 0.075 | 0.048 |
| 22S6 | 22 | 6 | Small | 0.075 | 0.048 |
| 22S7 | 22 | 7 | Small | 0.075 | 0.048 |
| 22S8 | 22 | 8 | Small | 0.075 | 0.048 |
| 24S1 | 24 | 1 | Small | 0.098 | 0.063 |
| 24S2 | 24 | 2 | Small | 0.098 | 0.063 |
| 24S3 | 24 | 3 | Small | 0.098 | 0.063 |
| 24S4 | 24 | 4 | Small | 0.098 | 0.063 |
| 24S5 | 24 | 5 | Small | 0.098 | 0.063 |
| 24S6 | 24 | 6 | Small | 0.098 | 0.063 |
| 25S1 | 25 | 1 | Small | 0.078 | 0.047 |
| 25S2 | 25 | 2 | Small | 0.078 | 0.047 |
| 25S3 | 25 | 3 | Small | 0.078 | 0.047 |
| 25S6 | 25 | 6 | Small | 0.078 | 0.047 |
| 25S7 | 25 | 7 | Small | 0.078 | 0.047 |
| 29S1 | 29 | 1 | Small | 0.026 | 0.017 |
| 29S2 | 29 | 2 | Small | 0.026 | 0.017 |
| 29S3 | 29 | 3 | Small | 0.026 | 0.017 |
| 29S4 | 29 | 4 | Small | 0.026 | 0.017 |
| 29S6 | 29 | 6 | Small | 0.026 | 0.017 |
| 29S7 | 29 | 7 | Small | 0.026 | 0.017 |
| 29S8 | 29 | 8 | Small | 0.026 | 0.017 |
| 3S1 | 3 | 1 | Small | 0.037 | 0.023 |
| 3S2 | 3 | 2 | Small | 0.037 | 0.023 |
| 3S3 | 3 | 3 | Small | 0.037 | 0.023 |
| 3S4 | 3 | 4 | Small | 0.037 | 0.023 |
| 3S5 | 3 | 5 | Small | 0.037 | 0.023 |
| 3S6 | 3 | 6 | Small | 0.037 | 0.023 |
| 3S8 | 3 | 8 | Small | 0.037 | 0.023 |
| 40S1 | 40 | 1 | Small | 0.028 | 0.013 |
| 40S2 | 40 | 2 | Small | 0.028 | 0.013 |
| 40S3 | 40 | 3 | Small | 0.028 | 0.013 |
| 40S4 | 40 | 4 | Small | 0.028 | 0.013 |
| 40S5 | 40 | 5 | Small | 0.028 | 0.013 |
| 40S6 | 40 | 6 | Small | 0.028 | 0.013 |
| 40S7 | 40 | 7 | Small | 0.028 | 0.013 |
| 40S8 | 40 | 8 | Small | 0.028 | 0.013 |
| 69S1 | 69 | 1 | Small | 0.032 | 0.018 |
| 69S2 | 69 | 2 | Small | 0.032 | 0.018 |
| 69S3 | 69 | 3 | Small | 0.032 | 0.018 |
| 69S4 | 69 | 4 | Small | 0.032 | 0.018 |
| 69S5 | 69 | 5 | Small | 0.032 | 0.018 |
| 69S6 | 69 | 6 | Small | 0.032 | 0.018 |
| 69S7 | 69 | 7 | Small | 0.032 | 0.018 |
| 74S1 | 74 | 1 | Small | 0.033 | 0.016 |
| 74S2 | 74 | 2 | Small | 0.033 | 0.016 |
| 74S3 | 74 | 3 | Small | 0.033 | 0.016 |
| 74S4 | 74 | 4 | Small | 0.033 | 0.016 |
| 74S6 | 74 | 6 | Small | 0.033 | 0.016 |
| 74S7 | 74 | 7 | Small | 0.033 | 0.016 |
| 74S8 | 74 | 8 | Small | 0.033 | 0.016 |
| 94S1 | 94 | 1 | Small | NA | NA |
| 94S2 | 94 | 2 | Small | NA | NA |
| 94S3 | 94 | 3 | Small | NA | NA |
| 94S4 | 94 | 4 | Small | NA | NA |
| 94S5 | 94 | 5 | Small | NA | NA |
| 94S6 | 94 | 6 | Small | NA | NA |
| 94S7 | 94 | 7 | Small | NA | NA |
| 94S8 | 94 | 8 | Small | NA | NA |
| 95S1 | 95 | 1 | Small | 0.03 | 0.018 |
| 95S2 | 95 | 2 | Small | 0.03 | 0.018 |
| 95S4 | 95 | 4 | Small | 0.03 | 0.018 |
| 95S6 | 95 | 6 | Small | 0.03 | 0.018 |
| 95S7 | 95 | 7 | Small | 0.03 | 0.018 |
| 95S8 | 95 | 8 | Small | 0.03 | 0.018 |

**Table S3**. Taxonomy and relative abundance of ASVs representing 99.9% of all rarefied reads. Non-taxonomic columns express total and average percentage of each ASV in all spat (n = 128), only large (n = 60), and only small (n = 68). ASVs highlighted in orange were differentially abundant in large spat, and ASVs highlighted in blue were differentially abundant in small spat. Taxonomy was assigned to the finest resolution possible.

| **ASV** | **Kingdom** | **Phylum** | **Class** | **Order** | **Family** | **Genus** | **Total % of reads in all** | **Avg. % of reads in all** | **Total % of reads in large** | **Avg. % of reads in large** | **Total % of reads in small** | **Avg. % of reads in small** |
| --- | --- | --- | --- | --- | --- | --- | --- | --- | --- | --- | --- | --- |
| asv01 | Bacteria | Tenericutes | Mollicutes | Mycoplasmatales | Mycoplasmataceae | *Mycoplasma* | 25.0691 | 24.4777 | 25.4265 | 25.8125 | 24.7464 | 23.3 |
| asv02 | Bacteria | Tenericutes | Mollicutes | Mycoplasmatales | Mycoplasmataceae | *Mycoplasma* | 6.5057 | 6.9986 | 7.9438 | 7.8232 | 5.2071 | 6.271 |
| asv03 | Bacteria | Proteobacteria | Alphaproteobacteria | Kordiimonadales | NA | NA | 3.8067 | 3.3126 | 3.3275 | 3.0767 | 4.2393 | 3.5207 |
| asv04 | Bacteria | Proteobacteria | Gammaproteobacteria | Cellvibrionales | Spongiibacteraceae | NA | 4.2091 | 3.7163 | 2.9073 | 2.8507 | 5.3845 | 4.48 |
| asv05 | Bacteria | Proteobacteria | Alphaproteobacteria | Rhodovibrionales | Kiloniellaceae | *Kiloniella* | 4.0392 | 3.7077 | 4.1964 | 3.964 | 3.8972 | 3.4815 |
| asv06 | Bacteria | Proteobacteria | Gammaproteobacteria | Oceanospirillales | Kangiellaceae | *Aliikangiella* | 3.2425 | 2.7315 | 3.914 | 3.3985 | 2.6361 | 2.143 |
| asv07 | Bacteria | Bacteroidetes | Bacteroidia | NA | NA | NA | 3.589 | 3.4505 | 3.6283 | 3.3914 | 3.5536 | 3.5027 |
| asv08 | Bacteria | Epsilonbacteraeota | Campylobacteria | Campylobacterales | Helicobacteraceae | NA | 3.7879 | 3.8056 | 3.8035 | 3.766 | 3.7738 | 3.8406 |
| asv09 | Bacteria | Proteobacteria | Gammaproteobacteria | Vibrionales | Vibrionaceae | NA | 2.9921 | 3.1248 | 4.2336 | 4.3716 | 1.871 | 2.0248 |
| asv10 | Bacteria | Proteobacteria | Alphaproteobacteria | Rhodospirillales | Rhodospirillaceae | NA | 2.6373 | 2.7415 | 5.2771 | 5.5643 | 0.2536 | 0.2508 |
| asv11 | Bacteria | Proteobacteria | Gammaproteobacteria | Alteromonadales | Psychromonadaceae | Psychromonas | 2.2894 | 2.4684 | 3.3954 | 3.751 | 1.2907 | 1.3367 |
| asv12 | Bacteria | Bacteroidetes | Bacteroidia | Flavobacteriales | NA | NA | 1.8992 | 1.7763 | 1.029 | 0.9621 | 2.6851 | 2.4948 |
| asv13 | Bacteria | Spirochaetes | Spirochaetia | Spirochaetales | Spirochaetaceae | NA | 1.7747 | 1.9778 | 2.5693 | 2.9547 | 1.0572 | 1.1159 |
| asv14 | Bacteria | Bacteroidetes | Bacteroidia | Flavobacteriales | Flavobacteriaceae | Aquimarina | 1.8744 | 1.7996 | 1.1989 | 1.2397 | 2.4844 | 2.2936 |
| asv15 | Bacteria | Proteobacteria | Gammaproteobacteria | NA | NA | NA | 1.5666 | 1.9567 | 0.1917 | 0.2424 | 2.808 | 3.4692 |
| asv16 | Bacteria | Proteobacteria | Gammaproteobacteria | Nitrosococcales | Methylophagaceae | NA | 1.1667 | 1.071 | 1.1393 | 1.0774 | 1.1914 | 1.0654 |
| asv17 | Bacteria | Proteobacteria | Gammaproteobacteria | Nitrosococcales | Methylophagaceae | NA | 1.0907 | 0.9908 | 0.9283 | 0.9765 | 1.2373 | 1.0034 |
| asv18 | Bacteria | Proteobacteria | Alphaproteobacteria | Kordiimonadales | Kordiimonadaceae | Kordiimonas | 1.146 | 1.0863 | 1.3513 | 1.2306 | 0.9606 | 0.9589 |
| asv19 | Bacteria | Bacteroidetes | Bacteroidia | Flavobacteriales | Flavobacteriaceae | Aquimarina | 1.1891 | 1.1639 | 0.5141 | 0.5379 | 1.7986 | 1.7162 |
| asv20 | Bacteria | Proteobacteria | Gammaproteobacteria | Alteromonadales | Colwelliaceae | Colwellia | 0.9573 | 0.9427 | 1.664 | 1.5978 | 0.3192 | 0.3646 |
| asv21 | Bacteria | Proteobacteria | Gammaproteobacteria | Oceanospirillales | Saccharospirillaceae | NA | 0.8483 | 0.7951 | 0.6523 | 0.6779 | 1.0253 | 0.8985 |
| asv22 | Bacteria | Proteobacteria | Alphaproteobacteria | Rhizobiales | Devosiaceae | NA | 0.9695 | 0.9367 | 1.4285 | 1.3453 | 0.555 | 0.5761 |
| asv23 | Bacteria | Proteobacteria | Gammaproteobacteria | Arenicellales | Arenicellaceae | NA | 0.8582 | 0.7309 | 0.7889 | 0.6716 | 0.9209 | 0.7832 |
| asv24 | Bacteria | Proteobacteria | Gammaproteobacteria | Nitrosococcales | Methylophagaceae | NA | 0.81 | 0.7511 | 0.885 | 0.8679 | 0.7423 | 0.648 |
| asv25 | Bacteria | Proteobacteria | Gammaproteobacteria | Vibrionales | Vibrionaceae | Vibrio | 0.7116 | 0.5177 | 1.1158 | 0.8148 | 0.3466 | 0.2555 |
| asv26 | Bacteria | Bacteroidetes | Ignavibacteria | OPB56 | NA | NA | 0.8312 | 0.8174 | 0.5604 | 0.5242 | 1.0758 | 1.076 |
| asv27 | Bacteria | Bacteroidetes | Bacteroidia | Chitinophagales | Saprospiraceae | NA | 0.7664 | 1.0008 | 0.362 | 0.4741 | 1.1315 | 1.4656 |
| asv28 | Bacteria | Proteobacteria | Alphaproteobacteria | Parvibaculales | NA | NA | 0.7978 | 0.7601 | 0.8302 | 0.7942 | 0.7685 | 0.7301 |
| asv29 | Bacteria | Proteobacteria | Alphaproteobacteria | Rhizobiales | Devosiaceae | Maritalea | 0.6324 | 0.6341 | 0.692 | 0.6675 | 0.5786 | 0.6046 |
| asv30 | Bacteria | Proteobacteria | Gammaproteobacteria | Oceanospirillales | Kangiellaceae | Aliikangiella | 0.6305 | 0.7226 | 0.3967 | 0.4537 | 0.8416 | 0.9598 |
| asv31 | Bacteria | Fusobacteria | Fusobacteriia | Fusobacteriales | Fusobacteriaceae | Psychrilyobacter | 0.744 | 0.6743 | 0.3326 | 0.2698 | 1.1154 | 1.0312 |
| asv32 | Bacteria | Proteobacteria | Gammaproteobacteria | Alteromonadales | Pseudoalteromonada-ceae | Pseudoalteromonas | 0.5273 | 0.5315 | 0.6393 | 0.5083 | 0.4262 | 0.5521 |
| asv33 | Bacteria | Bacteroidetes | Bacteroidia | Flavobacteriales | Flavobacteriaceae | Polaribacter | 0.5773 | 0.6553 | 0.6556 | 0.7947 | 0.5067 | 0.5323 |
| asv34 | Bacteria | Bacteroidetes | Bacteroidia | Flavobacteriales | Cryomorphaceae | NA | 0.5236 | 0.4882 | 0.7185 | 0.6557 | 0.3476 | 0.3405 |
| asv35 | Bacteria | Proteobacteria | Gammaproteobacteria | MBAE14 | NA | NA | 0.4807 | 0.5687 | 0.1134 | 0.1129 | 0.8123 | 0.9709 |
| asv36 | Bacteria | Proteobacteria | Alphaproteobacteria | Rhizobiales | Devosiaceae | Maritalea | 0.4884 | 0.4823 | 0.7293 | 0.6475 | 0.2709 | 0.3366 |
| asv37 | Bacteria | Proteobacteria | Alphaproteobacteria | Micavibrionales | Micavibrionaceae | NA | 0.3133 | 0.2343 | 0.179 | 0.1623 | 0.4346 | 0.2978 |
| asv38 | Bacteria | Proteobacteria | Deltaproteobacteria | Myxococcales | Nannocystaceae | NA | 0.32 | 0.2845 | 0.1525 | 0.1215 | 0.4712 | 0.4283 |
| asv39 | Bacteria | Proteobacteria | Deltaproteobacteria | Myxococcales | Nannocystaceae | NA | 0.4022 | 0.478 | 0.1187 | 0.1191 | 0.6582 | 0.7947 |
| asv40 | Bacteria | Proteobacteria | Alphaproteobacteria | Rhodobacterales | Rhodobacteraceae | NA | 0.427 | 0.5684 | 0.33 | 0.4582 | 0.5145 | 0.6655 |
| asv41 | Bacteria | Bacteroidetes | Bacteroidia | Flavobacteriales | Crocinitomicaceae | Salinirepens | 0.4014 | 0.4077 | 0.4927 | 0.4977 | 0.319 | 0.3283 |
| asv42 | Bacteria | Bacteroidetes | Bacteroidia | Flavobacteriales | Crocinitomicaceae | Crocinitomix | 0.4432 | 0.4472 | 0.219 | 0.2826 | 0.6457 | 0.5925 |
| asv43 | Bacteria | Proteobacteria | Deltaproteobacteria | Oligoflexales | Oligoflexaceae | NA | 0.4014 | 0.4131 | 0.0457 | 0.0527 | 0.7226 | 0.7311 |
| asv44 | Bacteria | NA | NA | NA | NA | NA | 0.4393 | 0.3456 | 0.8293 | 0.6207 | 0.0872 | 0.1028 |
| asv45 | Bacteria | Proteobacteria | Deltaproteobacteria | Myxococcales | Nannocystaceae | NA | 0.3366 | 0.3948 | 0.0965 | 0.0913 | 0.5534 | 0.6625 |
| asv46 | Bacteria | Proteobacteria | Alphaproteobacteria | Rhizobiales | Rhizobiaceae | Ahrensia | 0.3292 | 0.4216 | 0.2016 | 0.2699 | 0.4443 | 0.5555 |
| asv47 | Bacteria | Proteobacteria | Gammaproteobacteria | Vibrionales | Vibrionaceae | NA | 0.282 | 0.2388 | 0.5357 | 0.4181 | 0.0529 | 0.0807 |
| asv48 | Bacteria | Proteobacteria | Alphaproteobacteria | Rhizobiales | NA | NA | 0.2722 | 0.2441 | 0.3833 | 0.3549 | 0.1719 | 0.1463 |
| asv49 | Bacteria | Proteobacteria | Alphaproteobacteria | Parvibaculales | PS1_clade | NA | 0.3184 | 0.2967 | 0.1786 | 0.1672 | 0.4445 | 0.4109 |
| asv50 | Bacteria | Bacteroidetes | Bacteroidia | Flavobacteriales | Flavobacteriaceae | Aquibacter | 0.2504 | 0.3036 | 0.0747 | 0.1099 | 0.4091 | 0.4745 |
| asv51 | Bacteria | Epsilonbacteraeota | Campylobacteria | Campylobacterales | Arcobacteraceae | Arcobacter | 0.3387 | 0.3532 | 0.3787 | 0.3577 | 0.3026 | 0.3492 |
| asv52 | Bacteria | Proteobacteria | Alphaproteobacteria | Rhodobacterales | Rhodobacteraceae | Aliisedimentitalea | 0.2369 | 0.2785 | 0.1582 | 0.1842 | 0.308 | 0.3617 |
| asv53 | Bacteria | Bacteroidetes | Bacteroidia | Cytophagales | Cyclobacteriaceae | Ekhidna | 0.2657 | 0.3196 | 0.0635 | 0.0785 | 0.4483 | 0.5323 |
| asv54 | Bacteria | NA | NA | NA | NA | NA | 0.2881 | 0.2992 | 0.0785 | 0.0739 | 0.4774 | 0.498 |
| asv55 | Bacteria | Bacteroidetes | Bacteroidia | Flavobacteriales | Flavobacteriaceae | Aquimarina | 0.2564 | 0.3602 | 0.1244 | 0.1969 | 0.3755 | 0.5043 |
| asv56 | Bacteria | Proteobacteria | Gammaproteobacteria | Gammaproteobacteria_  Incertae_Sedis | Unknown_Family | Marinicella | 0.2751 | 0.3454 | 0.142 | 0.2232 | 0.3952 | 0.4533 |
| asv57 | Bacteria | Verrucomicrobia | Verrucomicrobiae | Verrucomicrobiales | Rubritaleaceae | Persicirhabdus | 0.2483 | 0.3004 | 0.0819 | 0.1016 | 0.3985 | 0.4758 |
| asv58 | Bacteria | Proteobacteria | Alphaproteobacteria | Rhizobiales | Stappiaceae | Labrenzia | 0.2285 | 0.2122 | 0.0844 | 0.0999 | 0.3586 | 0.3113 |
| asv59 | Bacteria | Epsilonbacteraeota | Campylobacteria | Campylobacterales | Arcobacteraceae | Arcobacter | 0.2444 | 0.2077 | 0.0296 | 0.0323 | 0.4384 | 0.3625 |
| asv60 | Bacteria | Proteobacteria | Alphaproteobacteria | Rhodobacterales | Rhodobacteraceae | Sulfitobacter | 0.2663 | 0.3641 | 0.069 | 0.0862 | 0.4445 | 0.6093 |
| asv61 | Bacteria | Proteobacteria | Deltaproteobacteria | Bdellovibrionales | Bacteriovoracaceae | Halobacteriovorax | 0.2362 | 0.2309 | 0.4056 | 0.3779 | 0.0832 | 0.1012 |
| asv62 | Bacteria | Proteobacteria | Alphaproteobacteria | Rhizobiales | Rhizobiaceae | Lentilitoribacter | 0.247 | 0.2954 | 0.369 | 0.4286 | 0.1368 | 0.1778 |
| asv63 | Archaea | Thaumarchaeota | Nitrososphaeria | Nitrosopumilales | Nitrosopumilaceae | Candidatus_  Nitrosopumilus | 0.1662 | 0.1724 | 0.0393 | 0.0377 | 0.2808 | 0.2912 |
| asv64 | Bacteria | Chloroflexi | Anaerolineae | SBR1031 | A4b | NA | 0.2164 | 0.1935 | 0.1999 | 0.1714 | 0.2313 | 0.213 |
| asv65 | Bacteria | Planctomycetes | Planctomycetacia | Pirellulales | Pirellulaceae | Blastopirellula | 0.1681 | 0.1767 | 0.0764 | 0.0776 | 0.2509 | 0.2642 |
| asv66 | Bacteria | Proteobacteria | Alphaproteobacteria | Kordiimonadales | NA | NA | 0.1864 | 0.2071 | 0.0493 | 0.0541 | 0.3103 | 0.3421 |
| asv67 | Bacteria | Epsilonbacteraeota | Campylobacteria | Campylobacterales | Arcobacteraceae | Arcobacter | 0.2295 | 0.1973 | 0.3292 | 0.2293 | 0.1396 | 0.1691 |
| asv68 | Bacteria | Proteobacteria | Gammaproteobacteria | Steroidobacterales | Woeseiaceae | NA | 0.1968 | 0.2748 | 0.0867 | 0.1202 | 0.2962 | 0.4112 |
| asv69 | Bacteria | Bacteroidetes | Bacteroidia | Cytophagales | Cyclobacteriaceae | Fabibacter | 0.1794 | 0.2098 | 0.0882 | 0.0982 | 0.2618 | 0.3082 |
| asv70 | Bacteria | Proteobacteria | Gammaproteobacteria | Oceanospirillales | Nitrincolaceae | Neptuniibacter | 0.1908 | 0.1804 | 0.0601 | 0.0435 | 0.3087 | 0.3012 |
| asv71 | Bacteria | Chloroflexi | Anaerolineae | SBR1031 | A4b | NA | 0.1612 | 0.1645 | 0.0375 | 0.0342 | 0.2729 | 0.2794 |
| asv72 | Bacteria | Epsilonbacteraeota | Campylobacteria | Campylobacterales | Helicobacteraceae | NA | 0.2035 | 0.2532 | 0.4265 | 0.538 | 0.0022 | 0.0019 |
| asv73 | Bacteria | Planctomycetes | Planctomycetacia | Planctomycetales | Rubinisphaeraceae | Rubinisphaera | 0.2019 | 0.1806 | 0.1301 | 0.1191 | 0.2668 | 0.2348 |
| asv74 | Bacteria | Tenericutes | Mollicutes | Mycoplasmatales | Mycoplasmataceae | Mycoplasma | 0.181 | 0.1851 | 0.3427 | 0.3682 | 0.0351 | 0.0236 |
| asv75 | Bacteria | Proteobacteria | Alphaproteobacteria | Rhodobacterales | Rhodobacteraceae | Maribius | 0.204 | 0.2771 | 0.1161 | 0.184 | 0.2834 | 0.3592 |
| asv76 | Bacteria | Proteobacteria | Alphaproteobacteria | Sphingomonadales | Sphingomonadaceae | Sphingorhabdus | 0.1691 | 0.2021 | 0.054 | 0.085 | 0.2729 | 0.3055 |
| asv77 | Bacteria | Proteobacteria | Gammaproteobacteria | Steroidobacterales | Woeseiaceae | Woeseia | 0.1546 | 0.2165 | 0.0127 | 0.0143 | 0.2827 | 0.3948 |
| asv78 | Bacteria | Actinobacteria | Acidimicrobiia | Microtrichales | Ilumatobacteraceae | Ilumatobacter | 0.1539 | 0.2139 | 0.135 | 0.2061 | 0.1709 | 0.2208 |
| asv79 | Bacteria | Proteobacteria | Gammaproteobacteria | Betaproteobacteriales | Nitrosomonadaceae | Nitrosomonas | 0.17 | 0.1641 | 0.073 | 0.0583 | 0.2575 | 0.2575 |
| asv80 | Bacteria | NA | NA | NA | NA | NA | 0.163 | 0.1594 | 0.0582 | 0.0932 | 0.2577 | 0.2178 |
| asv81 | Bacteria | Proteobacteria | Alphaproteobacteria | Parvibaculales | NA | NA | 0.1635 | 0.1391 | 0.1902 | 0.1585 | 0.1394 | 0.122 |
| asv82 | Bacteria | Proteobacteria | Alphaproteobacteria | Rhodospirillales | Terasakiellaceae | Terasakiella | 0.1714 | 0.1548 | 0.3174 | 0.2937 | 0.0396 | 0.0322 |
| asv83 | Bacteria | Verrucomicrobia | Verrucomicrobiae | Verrucomicrobiales | Rubritaleaceae | Persicirhabdus | 0.1509 | 0.1829 | 0.0408 | 0.0433 | 0.2503 | 0.3061 |
| asv84 | Bacteria | Firmicutes | Clostridia | Clostridiales | Family_XII | Fusibacter | 0.1703 | 0.1823 | 0.3539 | 0.3821 | 0.0046 | 0.0059 |
| asv85 | Bacteria | Bacteroidetes | Bacteroidia | Flavobacteriales | Flavobacteriaceae | Tenacibaculum | 0.1564 | 0.131 | 0.0442 | 0.0471 | 0.2577 | 0.205 |
| asv86 | Bacteria | Proteobacteria | Gammaproteobacteria | Oceanospirillales | Kangiellaceae | Aliikangiella | 0.15 | 0.1213 | 0.2131 | 0.1703 | 0.093 | 0.078 |
| asv87 | Bacteria | Proteobacteria | Gammaproteobacteria | Alteromonadales | Colwelliaceae | Thalassotalea | 0.1639 | 0.1642 | 0.0506 | 0.0521 | 0.2663 | 0.2632 |
| asv88 | Bacteria | Acidobacteria | Thermoanaerobaculia | Thermoanaerobaculales | Thermoanaerobacula-ceae | Subgroup_10 | 0.1601 | 0.1618 | 0.1413 | 0.1591 | 0.1771 | 0.1642 |
| asv89 | Bacteria | Proteobacteria | Deltaproteobacteria | Oligoflexales | Oligoflexaceae | NA | 0.1639 | 0.2269 | 0.3078 | 0.4388 | 0.0339 | 0.0399 |
| asv90 | Bacteria | Proteobacteria | Alphaproteobacteria | Rhodobacterales | Rhodobacteraceae | NA | 0.1426 | 0.2073 | 0.1062 | 0.1913 | 0.1755 | 0.2214 |
| asv91 | Bacteria | Proteobacteria | Alphaproteobacteria | Kordiimonadales | NA | NA | 0.1479 | 0.1513 | 0.1424 | 0.147 | 0.1529 | 0.1552 |
| asv92 | Bacteria | Proteobacteria | Gammaproteobacteria | Oceanospirillales | Kangiellaceae | Aliikangiella | 0.1303 | 0.1343 | 0.1663 | 0.1963 | 0.0978 | 0.0796 |
| asv93 | Bacteria | Proteobacteria | Alphaproteobacteria | Rhodospirillales | Magnetospiraceae | NA | 0.1329 | 0.1942 | 0.0453 | 0.077 | 0.212 | 0.2977 |
| asv94 | Bacteria | Proteobacteria | Alphaproteobacteria | Rhodobacterales | Rhodobacteraceae | Lentibacter | 0.1298 | 0.1896 | 0.0537 | 0.0892 | 0.1986 | 0.2782 |
| asv95 | Bacteria | Proteobacteria | Gammaproteobacteria | Pasteurellales | Pasteurellaceae | Haemophilus | 0.1679 | 0.4923 | 0 | 0 | 0.3195 | 0.9268 |
| asv96 | Bacteria | Proteobacteria | Alphaproteobacteria | Rhodobacterales | Rhodobacteraceae | Loktanella | 0.1425 | 0.1764 | 0.0567 | 0.0574 | 0.22 | 0.2815 |
| asv97 | Bacteria | Bacteroidetes | Bacteroidia | Chitinophagales | Chitinophagaceae | Vibrionimonas | 0.1618 | 0.3494 | 0.0933 | 0.1282 | 0.2236 | 0.5445 |

**Table S4**. Statistical analysis of alpha diversity measures. For Richness, phylogenetic distance, and Shannon index, assumptions of normality were met and students’ t-tests were run between the two categories of spat size (small and large). For Simpson’s index, data followed a beta distribution and a GLM was carried out to determine the significance of differences between the two size groups.

| **Measure** | **Size** | **Mean** | **SD** | **P-value** |
| --- | --- | --- | --- | --- |
| Richness | Large | 0.359 | 0.160 | 0.0001 |
|  | Small | 0.490 | 0.209 |  |
| Phylogenetic distance | Large | 0.414 | 0.183 | 0.002 |
|  | Small | 0.522 | 0.208 |  |
| Shannon | Large | 0.409 | 0.169 | 0.007 |
|  | Small | 0.510 | 0.243 |  |
| Simpson | Large | 0.724 | 0.201 | 0.53 |
|  | Small | 0.749 | 0.237 |  |

**Table S5**. ANOVA values for alpha diversity measures by oyster family and rarefied read depth. Oyster family did not have a significant effect on Shannon or Simpson index but did have a significant effect on phylogenetic distance and richness. Rarefied read depth did not impact diversity for Shannon or Simpson metrics but did impact phylogenetic distance and richness metrics.

| **Variable** | **Measure** | **P-value** |
| --- | --- | --- |
| Oyster family | Richness | 0.04 |
|  | Phylogenetic distance | < 0.001 |
|  | Shannon | 0.748 |
|  | Simpson | 0.39 |
| Rarefied read depth | Richness | < 0.0001 |
|  | Phylogenetic distance | < 0.0001 |
|  | Shannon | 0.354 |
|  | Simpson | 0.581 |

**Table S6**. Large core microbiome taxonomy and abundance. All ASVs belonged to the bacterial kingdom, so this taxonomic classification has been omitted. The seven ASVs unique to the large core microbiome (not present in the small core microbiome) are highlighted in gray.

| **ASV** | **Phylum** | **Class** | **Order** | **Family** | **Genus** | **Total % of large reads** | **Avg. % of large reads** |
| --- | --- | --- | --- | --- | --- | --- | --- |
| asv01 | Tenericutes | Mollicutes | Mycoplasmatales | Mycoplasmataceae | Mycoplasma | 25.4265 | 25.8125 |
| asv02 | Tenericutes | Mollicutes | Mycoplasmatales | Mycoplasmataceae | Mycoplasma | 7.9438 | 7.8232 |
| asv03 | Proteobacteria | Alphaproteobacteria | Kordiimonadales | NA | NA | 3.3275 | 3.0767 |
| asv04 | Proteobacteria | Gammaproteobacteria | Cellvibrionales | Spongiibacteraceae | NA | 2.9073 | 2.8507 |
| asv05 | Proteobacteria | Alphaproteobacteria | Rhodovibrionales | Kiloniellaceae | Kiloniella | 4.1964 | 3.964 |
| asv06 | Proteobacteria | Gammaproteobacteria | Oceanospirillales | Kangiellaceae | Aliikangiella | 3.914 | 3.3985 |
| asv08 | Epsilonbacteraeota | Campylobacteria | Campylobacterales | Helicobacteraceae | NA | 3.8035 | 3.766 |
| asv09 | Proteobacteria | Gammaproteobacteria | Vibrionales | Vibrionaceae | Aliivibrio | 4.2336 | 4.3716 |
| asv10 | Proteobacteria | Alphaproteobacteria | Rhodospirillales | Rhodospirillaceae | NA | 5.2771 | 5.5643 |
| asv11 | Proteobacteria | Gammaproteobacteria | Alteromonadales | Psychromonadaceae | Psychromonas | 3.3954 | 3.751 |
| asv12 | Bacteroidetes | Bacteroidia | Flavobacteriales | NA | NA | 1.029 | 0.9621 |
| asv13 | Spirochaetes | Spirochaetia | Spirochaetales | Spirochaetaceae | NA | 2.5693 | 2.9547 |
| asv18 | Proteobacteria | Alphaproteobacteria | Kordiimonadales | Kordiimonadaceae | Kordiimonas | 1.3513 | 1.2306 |
| asv20 | Proteobacteria | Gammaproteobacteria | Alteromonadales | Colwelliaceae | Colwellia | 1.664 | 1.5978 |
| asv22 | Proteobacteria | Alphaproteobacteria | Rhizobiales | Devosiaceae | NA | 1.4285 | 1.3453 |
| asv23 | Proteobacteria | Gammaproteobacteria | Arenicellales | Arenicellaceae | NA | 0.7889 | 0.6716 |
| asv26 | Bacteroidetes | Ignavibacteria | OPB56 | NA | NA | 0.5604 | 0.5242 |
| asv27 | Bacteroidetes | Bacteroidia | Chitinophagales | Saprospiraceae | NA | 0.362 | 0.4741 |
| asv28 | Proteobacteria | Alphaproteobacteria | Parvibaculales | NA | NA | 0.8302 | 0.7942 |
| asv29 | Proteobacteria | Alphaproteobacteria | Rhizobiales | Devosiaceae | Maritalea | 0.692 | 0.6675 |
| asv40 | Proteobacteria | Alphaproteobacteria | Rhodobacterales | Rhodobacteraceae | NA | 0.33 | 0.4582 |
| asv41 | Bacteroidetes | Bacteroidia | Flavobacteriales | Crocinitomicaceae | Salinirepens | 0.4927 | 0.4977 |
| asv42 | Bacteroidetes | Bacteroidia | Flavobacteriales | Crocinitomicaceae | Crocinitomix | 0.219 | 0.2826 |
| asv46 | Proteobacteria | Alphaproteobacteria | Rhizobiales | Rhizobiaceae | Ahrensia | 0.2016 | 0.2699 |
| asv62 | Proteobacteria | Alphaproteobacteria | Rhizobiales | Rhizobiaceae | Lentilitoribacter | 0.369 | 0.4286 |
| asv89 | Proteobacteria | Deltaproteobacteria | Oligoflexales | Oligoflexaceae | NA | 0.3078 | 0.4388 |

**Table S7**. Small core microbiome taxonomy and abundance. All ASVs belonged to the bacterial kingdom, so this taxonomic classification has been omitted. The 16 ASVs unique to the small core microbiome (not present in the large core microbiome) are highlighted in gray.

| **ASV** | **Phylum** | **Class** | **Order** | **Family** | **Genus** | **Total % of small reads** | **Avg. % of small reads** |
| --- | --- | --- | --- | --- | --- | --- | --- |
| asv01 | Tenericutes | Mollicutes | Mycoplasmatales | Mycoplasmataceae | Mycoplasma | 24.7464 | 23.3 |
| asv02 | Tenericutes | Mollicutes | Mycoplasmatales | Mycoplasmataceae | Mycoplasma | 5.2071 | 6.271 |
| asv03 | Proteobacteria | Alphaproteobacteria | Kordiimonadales | NA | NA | 4.2393 | 3.5207 |
| asv04 | Proteobacteria | Gammaproteobacteria | Cellvibrionales | Spongiibacteraceae | NA | 5.3845 | 4.48 |
| asv05 | Proteobacteria | Alphaproteobacteria | Rhodovibrionales | Kiloniellaceae | Kiloniella | 3.8972 | 3.4815 |
| asv06 | Proteobacteria | Gammaproteobacteria | Oceanospirillales | Kangiellaceae | Aliikangiella | 2.6361 | 2.143 |
| asv08 | Epsilonbacteraeota | Campylobacteria | Campylobacterales | Helicobacteraceae | NA | 3.7738 | 3.8406 |
| asv09 | Proteobacteria | Gammaproteobacteria | Vibrionales | Vibrionaceae | Aliivibrio | 1.871 | 2.0248 |
| asv11 | Proteobacteria | Gammaproteobacteria | Alteromonadales | Psychromonadaceae | Psychromonas | 1.2907 | 1.3367 |
| asv12 | Bacteroidetes | Bacteroidia | Flavobacteriales | NA | NA | 2.6851 | 2.4948 |
| asv13 | Spirochaetes | Spirochaetia | Spirochaetales | Spirochaetaceae | NA | 1.0572 | 1.1159 |
| asv15 | Proteobacteria | Gammaproteobacteria | NA | NA | NA | 2.808 | 3.4692 |
| asv18 | Proteobacteria | Alphaproteobacteria | Kordiimonadales | Kordiimonadaceae | Kordiimonas | 0.9606 | 0.9589 |
| asv19 | Bacteroidetes | Bacteroidia | Flavobacteriales | Flavobacteriaceae | Aquimarina | 1.7986 | 1.7162 |
| asv22 | Proteobacteria | Alphaproteobacteria | Rhizobiales | Devosiaceae | NA | 0.555 | 0.5761 |
| asv26 | Bacteroidetes | Ignavibacteria | OPB56 | NA | NA | 1.0758 | 1.076 |
| asv27 | Bacteroidetes | Bacteroidia | Chitinophagales | Saprospiraceae | NA | 1.1315 | 1.4656 |
| asv28 | Proteobacteria | Alphaproteobacteria | Parvibaculales | NA | NA | 0.7685 | 0.7301 |
| asv30 | Proteobacteria | Gammaproteobacteria | Oceanospirillales | Kangiellaceae | Aliikangiella | 0.8416 | 0.9598 |
| asv35 | Proteobacteria | Gammaproteobacteria | MBAE14 | NA | NA | 0.8123 | 0.9709 |
| asv40 | Proteobacteria | Alphaproteobacteria | Rhodobacterales | Rhodobacteraceae | NA | 0.5145 | 0.6655 |
| asv41 | Bacteroidetes | Bacteroidia | Flavobacteriales | Crocinitomicaceae | Salinirepens | 0.319 | 0.3283 |
| asv46 | Proteobacteria | Alphaproteobacteria | Rhizobiales | Rhizobiaceae | Ahrensia | 0.4443 | 0.5555 |
| asv50 | Bacteroidetes | Bacteroidia | Flavobacteriales | Flavobacteriaceae | Aquibacter | 0.4091 | 0.4745 |
| asv53 | Bacteroidetes | Bacteroidia | Cytophagales | Cyclobacteriaceae | Ekhidna | 0.4483 | 0.5323 |
| asv54 | NA | NA | NA | NA | NA | 0.4774 | 0.498 |
| asv56 | Proteobacteria | Gammaproteobacteria | Gammaproteobacteria_  Incertae_Sedis | Unknown_Family | Marinicella | 0.3952 | 0.4533 |
| asv57 | Verrucomicrobia | Verrucomicrobiae | Verrucomicrobiales | Rubritaleaceae | Persicirhabdus | 0.3985 | 0.4758 |
| asv60 | Proteobacteria | Alphaproteobacteria | Rhodobacterales | Rhodobacteraceae | Sulfitobacter | 0.4445 | 0.6093 |
| asv68 | Proteobacteria | Gammaproteobacteria | Steroidobacterales | Woeseiaceae | NA | 0.2962 | 0.4112 |
| asv69 | Bacteroidetes | Bacteroidia | Cytophagales | Cyclobacteriaceae | Fabibacter | 0.2618 | 0.3082 |
| asv73 | Planctomycetes | Planctomycetacia | Planctomycetales | Rubinisphaeraceae | Rubinisphaera | 0.2668 | 0.2348 |
| asv76 | Proteobacteria | Alphaproteobacteria | Sphingomonadales | Sphingomonadaceae | Sphingorhabdus | 0.2729 | 0.3055 |
| asv78 | Actinobacteria | Acidimicrobiia | Microtrichales | Ilumatobacteraceae | Ilumatobacter | 0.1709 | 0.2208 |
| asv93 | Proteobacteria | Alphaproteobacteria | Rhodospirillales | Magnetospiraceae | NA | 0.212 | 0.2977 |

**Table S8**. The 19 differentially abundant ASVs and the ANCOM-BC2 output statistics associated with them. All differentially abundant ASVs belonged to the bacterial kingdom, so this taxonomic classification has been omitted. Taxa differentially abundant in the large spat are highlighted in orange, while those more abundant in small spat are in blue. LFC, log fold change in abundance in large over small; SE, standard error of LFC; W, test statistic (LFC divided by the standard deviation); q-value, significance after Benjimini Hochberg p-value correction in DESeq2 test. Taxonomy was assigned to finest resolution possible.

| **ASV** | **LFC** | **SE** | **W** | **q-value** | **Phylum** | **Class** | **Order** | **Family** | **Genus** |
| --- | --- | --- | --- | --- | --- | --- | --- | --- | --- |
| asv01 | 1.131 | 0.433 | 2.611 | 0.046 | Tenericutes | Mollicutes | Mycoplasmatales | Mycoplasmataceae | *Mycoplasma* |
| asv06 | 1.363 | 0.527 | 2.587 | 0.046 | Proteobacteria | Gammaproteobacteria | Oceanospirillales | Kangiellaceae | *Aliikangiella* |
| asv09 | 1.426 | 0.457 | 3.118 | 0.015 | Proteobacteria | Gammaproteobacteria | Vibrionales | Vibrionaceae | NA |
| asv10 | 3.409 | 0.490 | 6.964 | < 0.001 | Proteobacteria | Alphaproteobacteria | Rhodospirillales | Rhodospirillaceae | NA |
| asv11 | 1.609 | 0.454 | 3.547 | 0.006 | Proteobacteria | Gammaproteobacteria | Alteromonadales | Psychromonadaceae | *Psychromonas* |
| asv13 | 1.969 | 0.502 | 3.918 | 0.002 | Spirochaetes | Spirochaetia | Spirochaetales | Spirochaetaceae | NA |
| asv20 | 1.635 | 0.502 | 3.256 | 0.010 | Proteobacteria | Gammaproteobacteria | Alteromonadales | Colwelliaceae | *Colwellia* |
| asv22 | 1.563 | 0.457 | 3.424 | 0.008 | Proteobacteria | Alphaproteobacteria | Rhizobiales | Devosiaceae | NA |
| asv29 | 1.351 | 0.474 | 2.853 | 0.026 | Proteobacteria | Alphaproteobacteria | Rhizobiales | Devosiaceae | *Maritalea* |
| asv35 | -1.650 | 0.442 | -3.734 | 0.003 | Proteobacteria | Gammaproteobacteria | MBAE14 | NA | NA |
| asv41 | 1.236 | 0.443 | 2.788 | 0.030 | Bacteroidetes | Bacteroidia | Flavobacteriales | Crocinitomicaceae | *Salinirepens* |
| asv48 | 1.094 | 0.420 | 2.605 | 0.046 | Proteobacteria | Alphaproteobacteria | Rhizobiales | NA | NA |
| asv53 | -1.279 | 0.427 | -2.998 | 0.019 | Bacteroidetes | Bacteroidia | Cytophagales | Cyclobacteriaceae | *Ekhidna* |
| asv60 | -1.321 | 0.442 | -2.993 | 0.019 | Proteobacteria | Alphaproteobacteria | Rhodobacterales | Rhodobacteraceae | *Sulfitobacter* |
| asv61 | 1.206 | 0.410 | 2.940 | 0.021 | Proteobacteria | Deltaproteobacteria | Bdellovibrionales | Bacteriovoracaceae | *Halobacteriovorax* |
| asv62 | 1.490 | 0.450 | 3.308 | 0.009 | Proteobacteria | Alphaproteobacteria | Rhizobiales | Rhizobiaceae | *Lentilitoribacter* |
| asv72 | 1.910 | 0.400 | 4.776 | < 0.001 | Epsilonbacteraeota | Campylobacteria | Campylobacterales | Helicobacteraceae | NA |
| asv82 | 1.340 | 0.396 | 3.383 | 0.008 | Proteobacteria | Alphaproteobacteria | Rhodospirillales | Terasakiellaceae | *Terasakiella* |
| asv89 | 2.072 | 0.397 | 5.223 | < 0.001 | Proteobacteria | Deltaproteobacteria | Oligoflexales | Oligoflexaceae | NA |
